# Circular RNA circERBB2 promotes gallbladder cancer progression by regulating PA2G4-dependent rDNA transcription

**DOI:** 10.1186/s12943-019-1098-8

**Published:** 2019-11-21

**Authors:** Xince Huang, Ming He, Shuai Huang, Ruirong Lin, Ming Zhan, Dong Yang, Hui Shen, Sunwang Xu, Wei Cheng, Jianxiu Yu, Zilong Qiu, Jian Wang

**Affiliations:** 1 Department of Biliary-Pancreatic Surgery, Renji Hospital, School of Medicine, Shanghai Jiao Tong University, 160 Pujian Road, Shanghai, 200127 People’s Republic of China; 2 Basic Research Laboratory, Department of Biliary-Pancreatic Surgery, Renji Hospital, School of Medicine, Shanghai Jiao Tong University, 160 Pujian Road, Shanghai, 200127 People’s Republic of China; 3 Department of Biochemistry and Molecular Cell Biology, Shanghai Key Laboratory of Tumor Microenvironment and Inflammation, Shanghai Jiao Tong University School of Medicine, Shanghai, 200025 China; 4 Institute of Neuroscience, State Key Laboratory of Neuroscience, CAS Center for Excellence in Brain Science and Intelligence Technology, University of Chinese Academy of Sciences, Chinese Academy of Sciences, Shanghai, 200031 People’s Republic of China

**Keywords:** Circular RNA, circERBB2, Gallbladder cancer, rDNA, PA2G4

## Abstract

**Background:**

CircRNAs are found to affect initiation and progression of several cancer types. However, whether circRNAs are implicated in gallbladder cancer (GBC) progression remains obscure.

**Methods:**

We perform RNA sequencing in 10 pairs of GBC and para-cancer tissues. CCK8 and clone formation assays are used to evaluate proliferation ability of GBC cells. qPCR and Western blot are used to determine expression of RNAs and proteins, respectively. CircRNA-protein interaction is confirmed by RNA pulldown, RNA immunoprecipitation, and fluorescence in situ hybridization.

**Results:**

We find that circRNA expression pattern is tremendously changed during GBC development. Among dozens of significantly changed circRNAs, a circRNA generated from the oncogene ERBB2, named as circERBB2, is one of the most significant changes. CircERBB2 promotes GBC proliferation, in vitro and in vivo. Other than being a miRNA sponge, circERBB2 accumulates in the nucleoli and regulates ribosomal DNA transcription, which is one of the rate-limiting steps of ribosome synthesis and cellular proliferation. CircERBB2 regulates nucleolar localization of PA2G4, thereby forming a circERBB2-PA2G4-TIFIA regulatory axis to modulate ribosomal DNA transcription and GBC proliferation. Increased expression of circERBB2 is associated with worse prognosis of GBC patients.

**Conclusions:**

Our findings demonstrate that circERBB2 serves as an important regulator of cancer cell proliferation and shows the potential to be a new therapeutic target of GBC.

## Background

Gallbladder cancer (GBC) is the most common bile tract malignancy with high malignancy and late presentation [1]. Surgical resection can achieve radical cure in early stage patients, but when GBC patients are symptomatic, there is no effective treatment [2]. Five-year survival rate is < 10% for stage-III patients and < 5% for stage-IV patients [3]. Although obvious improvement has been made in the diagnosis, operation, and adjuvant therapy, little impact has been made on overall survival.

The development of high throughput sequencing has verified that circular RNA (circRNA) is an entire class of abundant, non-coding RNAs ubiquitous among eukaryotes [4–6]. CircRNAs are generated when the precursor mRNAs (pre-mRNA) back-splice to join a downstream splice donor to an upstream splice acceptor. Recent studies show that circRNAs play critical roles in cellular differentiation, neural development, and disease occurrence [7–9]. Though most functional circRNAs are reported to work as miRNA sponges, only few circRNAs contain multiple binding sites to trap one specific miRNA [5]. This indicates that some circRNAs may function in ways beyond miRNA sponge.

Many circRNAs have been implicated in cancer initiation and progression. For examples, circITCH and circZNF292 affect cancer growth by targeting Wnt/β-catenin signaling [10, 11]. CircFoxo3 has been implicated in inhibition of cell cycle progression by forming a circFoxo3-p21-CDK2 ternary complex, which prevents CDK2 from promoting cell cycle progression [12]. Elevated circCCDC66 in colon polyps and colon cancer controls invasion and migration of colorectal cancer cells [13]. Moreover, some other circRNAs, like circNRIP1, circCSNK1G3, and circFAT1, affect several of the hallmarks of cancer [14–16]. On the other hand, due to lack of the open end, circRNAs are resistant to exoribonuclease treatment and can stably exist in some body fluids like serum, saliva, and even urine [17]. This makes circRNAs novel biomarkers for cancer diagnosis and prognosis from non-invasive clinical samples [17–19].

The active ribosomal DNA (rDNA) is transcribed by RNA polymerase I (Pol I) to produce pre-ribosomal RNA (rRNA), which is named as 45S and processed to yield 5.8S, 18S and 28S rRNA. Cells undergoing malignant proliferation are dependent on a concomitant increased rate of protein synthesis, which in turn, is dependent on synthesis of ribosome. Thus, ribosome synthesis is a rate-limiting step for malignant cellular proliferation [20]. Cancer cells, which are hallmarked by enlarged nucleoli, often have abnormal rDNA transcription by Pol I [21, 22]. The rDNA transcription and Pol I activity are regulated by many factors including some proteins, non-coding RNAs and epigenetic alterations [23–26]. Many factors associated with rDNA transcription are related with malignant transformation and cancer progression, and rRNA synthesis has been recognized as a potential target for cancer therapeutic intervention [21, 23].

Here, we report the function and clinical implication of a circRNA derived from ERBB2 gene locus, referred to circERBB2. Differing from general circRNAs that are located in cytoplasm and function as miRNA sponges, circERBB2 is enriched in nucleoli, up-regulates Pol I activity and rDNA transcription, finally promotes GBC proliferation and progression. CircERBB2 has important clinical implications for GBC patients.

## Methods

### Patients and samples

All surgically removed primary GBC and para-cancer specimens were obtained from Biliary-Pancreatic Surgery Department of Shanghai Renji Hospital, with informed consent. Specimens were collected in accordance with institutional protocols. Ten pairs of cancer and corresponding para-cancer samples were selected for RNA sequencing, 29 pairs were selected for RNA and protein extraction (and only 28 pairs were succeeded in protein extraction due to the limitation of tumor size), and 149 cancer samples were embedded with paraffin to make the tissue microarray.

### Cell culture and transfection

GBC cell lines SGC-996 and GBC-SD and human embryonic kidney 293 T cells were cultured in high glucose DMEM medium with 10% fetal bovine serum, 100 U/ml penicillin, and 100 μg/ml streptomycin.

Lipofectamine 2000 and polyethyleneimine were used for transfection of GBC cells and 293 T cells respectively, according to the manufacturer’s protocols.

### Silencing of gene expression by siRNAs

CircERBB2, PA2G4 and TIFIA were silenced by at least 2 siRNAs. Sequences of siRNAs were listed in Additional file 1: Table S1. For siRNA transfection, siRNAs were mixed with Lipofectamine 2000 with final concentration of 50 μM. Total protein or RNA was collected 48 h later to determine effects of siRNAs.

### Plasmids

#### pLenti-CMV-circERBB2

Full length gDNA from intron 2 (− 791 bp from back-splicing site) to intron 7 (+ 1342 bp from back-splicing site) of ERBB2 gene was PCR amplified from the gDNA of SGC-996 cells. pLenti-CMV-EGFP-3Flag vector was treated with EcoRI and XbaI restriction endonuclease to remove EGFP-3Flag. Then intron 2 to intron 7 gDNA of ERBB2 was cloned into the vector with T4 DNA-ligase. Back-splicing between exon 3 and exon 7 was mediated by sequence of flanking introns.

#### pLenti-CMV-PA2G4-Flag

Full length PA2G4 cDNA was amplified from cDNA of 293 T cells and cloned into pLenti-CMV-EGFP-3Flag vector using EcoRI and BamHI.

#### pLenti-CMV-PA2G4-mCherry-Flag

pLenti-CMV-mCherry-3Flag vector was treated with EcoRI for linearization. Full length PA2G4 cDNA was amplified from cDNA of 293 T cells and cloned into the linearized vector using seamless clone kit (Beyotime). Then PA2G4 cDNA with mutant C-terminal polybasic region was obtained by overlap extension PCR and cloned into the linearized vector using seamless clone kit.

#### pLenti-CMV-TIFIA-Flag

Full length TIFIA cDNA was amplified from cDNA of 293 T cells and cloned into pLenti-CMV-EGFP-3Flag vector using EcoRI and BamHI.

#### pGL3-rDNA-IRES

The rDNA promoter luciferase reporter vector was generously given by Chen Ling-Ling group of Shanghai Institute of Biochemistry and Cell Biology, Chinese Academy of Sciences [25].

### RNA isolation, reverse transcription PCR (rt-PCR) and qPCR

Total RNA from cells or tissue samples were extracted with Trizol Reagent (Sigma) according to the manufacturer’s protocol. First strand cDNA was synthesized with RevertAid First Strand cDNA Synthesis Kit (Thermo) according to the manufacturer’s protocol. qPCR was done with SybrGreen PCR Master Mix (Yeasen) and Quantstudio™ DX Real-Time PCR Instrument (ABI). The relative gene expression was quantified to GAPDH or 18S. Primers used for qPCR were listed in Additional file 1: Table S1.

### RNase R resistance assay

Five micrograms total RNA extracted from SGC-996 cells was incubated with RNase R at 37 °C for 10 min. As control, another 5 μg total RNA was incubated with RNase-free water at the same condition. RNase-free water was added to a total volume of 11 μl. After incubation, 1 μl random primer from RevertAid First Strand cDNA Synthesis Kit was added to the mix to start rt-PCR.

### CircERBB2 copy number measurement

PCR products of divergent primers targeting back-splicing sequence of circERBB2 and CDR1as and convergent primers targeting ERBB2 mRNA were cloned into pLenti-CMV-EGFP-Flag vector. Then the plasmids were linearized with EcoRI and serial diluted as template for qPCR to generate a standard curve for circERBB2, CDR1as, and ERBB2 mRNA. Molecular weight of the plasmids was calculated as 660 multiply base number. Total RNA from 10^6^ SGC-996 and GBC-SD cells was extracted and 1/10, which represents total RNA of 10^5^ cells, was used for rt-PCR and qPCR. Copy number was calculated according to the standard curve.

### Cell counting kit-8 (CCK8) and clone formation assay

GBC cell lines were seeded in 96-well plate at concentration of 2000/well. One hundred microliters FBS-free medium with 10% CCK8 reagent was added to the corresponding wells and incubated at 37 °C for 2 h. Absorbance was measured with microplate reader (Biotech). A well without cell was used to determine basic absorbance. Cell viability-related absorbance was equal to measured absorbance minus basic absorbance.

For clone formation assay, 500/well of GBC cells were seeded into 6-well plate and cultured at 37 °C for 14 days. Then cells were fixed with methanol and stained with crystal violet.

### RNA pulldown

CircRNA pulldown was performed using Magnetic RNA-protein Pull-down Kit (Thermo), with some modifications of manufacturer’s protocol. We designed desthiobiotin-labeld DNA probe (Additional file 1: Table S1) targeting back-splicing sequence of circERBB2 to effectively and specifically capture circERBB2 from total RNA. 10^7^ 293 T cells were transfected with circERBB2 over-expression (OE) vector or control vector. 48 h later, total RNA from the two groups was extracted and incubated with 100 nmol DNA probe respectively at 70 °C for 5 min. Then RNA was slowly cooled down to room temperature and circERBB2 would hybridize with probe. Then 50 μl Streptavidin Magnetic Beads was added and incubated at room temperature for 30 min with agitation. Unbound RNA was washed away by 20 mM Tris, and 100 μl 1 × RNA-protein binding buffer with 100 μg total protein was added to the tube containing Streptavidin Magnetic Beads. After incubation at 4 °C for 1.5 h with rotation, Streptavidin Magnetic Beads were washed with washing buffer for 3 times, and then incubated with 50 μl Elution buffer at 37 °C for 15 min with agitation. Supernatant was collected for mass spectrum or western blot.

### Silver staining

After RNA pulldown, equal amount of protein was loaded to 10% polypropylene gel. After 1.5 h of electrophoresis, silver staining was performed with Silver stain kit (Beyotime) according to manufacturer’s instruction.

### RNA-protein immunoprecipitation (RIP)

293 T cells were cultured in two 15 cm dish and transfected with pLenti-CMV-EGFP-Flag or pLenti-CMV-PA2G4-Flag expression vectors respectively. 48 h later, about 2 × 10^7^ cells in each dish were harvested and resuspended in 250 μl lysis buffer [20 mM Tris (8.0), 150 mM NaCl, 10% glycerol, 1% NP-40, 2 mM EDTA, 0.5 mM PMSF, protease inhibitor cocktail, 2 mM Ribonucleoside Vanadyl Complexes (RVC), 2 μl RNase inhibitor (Beyotime)]. 1/10 volume of the lysates were transferred to another two RNase-free tubes and labeled as input, and the rest lysates were used for RIP assay. All lysates were frozen immediately at − 80 °C overnight to gently lyse the cells. Then thawed the RIP lysates quickly and centrifuged at 15,000 rpm for 10 min at 4 °C. The supernatant was collected and incubated with 50 μl Anti-Flag Beads at 4 °C for 3 h with rotation. The Flag-Beads were washed with 500 μl lysis buffer for 5 times. After last washing, thawed the input lysates and added 1 ml Trizol to the input lysates and the Flag-Beads to extract total RNA. Input and immunoprecipitated RNA were reverse transcribed and qPCR was used to determine enrichment of targeted RNA.

### Knock-down of circERBB2 by CRISPR-cas9

Two sgRNA individually targeting boundaries of the Alu repeat in ERBB2 intron 7 were inserted into PX330 plasmid. Same amount of the two plasmids were transfected into SGC-996 and GBC-SD cells. 48 h later, about 2000 cells were seeded in a 10 cm dish. When clones were visible with naked eyes, about 50–100 clones of each cell line were collected and seeded into 12-well plates (each well contains one clone). When number of cells reached about 10^6^, half of cells were harvested and treated with protease K at 55 °C for 1 h and 95 °C for 15 min. Lysates were used as template for PCR with primer pair targeting boundaries of the sgRNA targeted region. Sequence of the targeted gDNA region was inferred from length of PCR fragment and confirmed by DNA sequencing.

### Dual luciferase reporter assay

pGL3-rDNA-IRES and Renilla luciferase vector (5:1) were co-transfected into 293 T cells. Renilla luciferase vector was used as an internal control for normalization. 48 h later, the luciferase activity was measured using Dual Luciferase Reporter Assay Kit (Yeasen) according to manufacturer’s protocols.

### Nucleoli isolation

For nucleolar protein extraction, 10^7^ SGC-996 cells were harvested and washed with PBS for 3 times. 1/10 cells were transferred to another tube and incubated with RIPA buffer on ice for 30 min. And then the lysate was centrifuged at 15,000 rpm for 10 min at 4 °C. The supernatant was collected as total protein lysate. The rest cells for nucleoli isolation were lysed with 100 μl lysis buffer [10 mM HEPES pH 7.9, 1.5 mM MgCl_2_, 10 mM KCl, 0.5 mM DTT, and 0.5 mM PMSF] on ice for 20 min. The lysate was centrifuge at 1000 rpm for 5 min at 4 °C and the supernatant was discarded. Nuclei pellet in the tube bottom was resuspended with 200 μl, 340 mM sucrose solution containing 5 mM MgCl_2_. After broken by sonication, 200 μL, 880 mM sucrose solution containing 5 mM MgCl_2_ was gently added to sonicated nuclei and then centrifuged at 2000 rpm, 4 °C for 20 min to pellet nucleoli. Supernatant was discarded and nucleoli pellet was lysed by RIPA buffer.

To extract nucleolar RNA, 10^7^ SGC-996 cells were harvested and 1/10 cells were transferred to another tube for total RNA extraction. The rest cells were lysed with 100 μl lysis buffer [10 mM HEPES pH 7.9, 1.5 mM MgCl_2_, 10 mM KCl, 0.5 mM DTT, 2 mM RVC, and 2 μl RNase inhibitor] on ice for 20 min. Then the lysate was centrifuge at 1000 rpm for 5 min at 4 °C and the supernatant was collected for cytoplasm RNA extraction. 1/10 nuclei pellet were transferred to another tube for nuclear RNA extraction. The rest nuclei pellet was resuspended, broken by sonication, and centrifuged as described above. Nucleoli pellet was washed with 340 mM sucrose solution containing 5 mM MgCl_2_ once. Then all lysates were treated with Trizol to extract RNA.

### Fluorescence in situ hybridization (FISH)

Paraffin sections were incubated at 62 °C for 1 h, de-waxed with xylene, and then hydrated. The slides of cultured cells were fixed with 4% paraformaldehyde. Then slides were incubated with proteinase K (20 mg/ml) for 10 min at room temperature, followed by incubation of 0.07% streptavidin solution at 37 °C to block endogenous biotin. Then slides were incubated with pre-hybrid buffer [2 × SSC, 50% formamide, 10 × Denhardt, 0.5% SDS, 10 mM sodium phosphate buffer (pH 6.6), 40 μg/ml denatured sperm DNA] at 37 °C for 1 h. After pre-hybridization, slides were incubated with pre-hybrid buffer with 0.1 μg biotin-labeled probe at 33 °C overnight and washed with 2 × SSC (37 °C, 10 min) and 1 × SSC (37 °C, 5 min, 2 times) in order. Then slides were incubated with FITC-labeled streptavidin (1:400) at room temperature, washed with PBS and stained with DAPI.

### FISH + immunofluorescence (IF) double staining

The slides of cultured cells were fixed with 4% paraformaldehyde, permeabilized with 1% Triton X-100, blocked with 0.07% streptavidin solution, pre-hybridized and hybridized as FISH assay. Then slides were incubated with anti-PA2G4, anti-NCL or anti-TIFIA antibody at 4 °C overnight, followed by incubation of Alexa Fluor 594 conjugated secondary antibody at 37 °C for 1 h. Then slides were incubated with FITC-labeled streptavidin (1:400) at room temperature, washed with PBS and stained with DAPI.

### IF and dual-IF staining

2 × 10^5^ SGC-996 were seeded in a sterile coverslip. After adherence, cells were fixed with 4% paraformaldehyde, incubated with 1% Triton X-100 and blocked with secondary antibody homologous serum. Then cells were incubated with 1:200 anti-PA2G4 antibody at 4 °C overnight, washed with PBS and incubated with Alexa Fluor 594 conjugated secondary antibody (1:250) at 37 °C for 1 h. Nuclei were stained with DAPI.

For dual-IF staining, cells were incubated with 1:200 anti-PA2G4 antibody and 1:100 anti-TIFIA antibody at 4 °C overnight, washed with PBS and incubated with Alexa Fluor 594 and Alexa Fluor 488 conjugated secondary antibody (1:250) at 37 °C for 1 h.

### Western blot and immunohistochemistry (IHC)

Western blot and IHC were performed as previously described [27, 28].

### Tumor xenografts

2 × 10^6^ GBC-SD cells were resuspended in 100 μl PBS and injected subcutaneously into flanks of 5-week-old male C57BL6 nude mice (*n* = 10). Six weeks later, mice were sacrificed by excessive chloral hydrate injection. Xenografts were integrally removed, imaged and fixed with 4% paraformaldehyde. Weight of xenografts was used for quantitative analysis of in vivo growth of GBC cells [29].

### Statistical analysis

Data were presented as the mean ± SD. Paired or non-paired 2-tailed t-test was used for comparisons between 2 groups. Pearson correlation coefficient and linear regression model were used to evaluate correlation between expression of circRNA and homogenous mRNA, circRNA and homogenous circRNA, or circERBB2 and PA2G4. BH method was used for FDR calculation. R-3.4 was used for statistical analysis. *p* < 0.05 was considered significant.

## Results

### Different circRNA expression patterns between GBC and para-cancer tissues

Through RNA sequencing of 10 pairs of GBC and para-cancer tissues, we totally identified 48,304 distinct circRNA candidates with at least 2 unique back-splicing reads. We used back-splicing reads per million reads (BRPM) to evaluate expression levels of circRNAs. The expression levels of different circRNAs varied dramatically, with BRPM values ranged from less than 0.1 to more than 100 (Additional file 2: Figure S1a). Most circRNAs were expressed at very low level, with more than 95% circRNAs had BRPM< 1 (Additional file 2: Figure S1b). Meanwhile, some were significantly higher than the average value with BRPM> 10 or even 100, and among them were some canonical genes like circHIPK3 and CDR1as [8, 30]. Most circRNAs were detected only in a fraction of the 20 samples, whereas some were widely expressed in most samples and appeared to have higher expression levels (Additional file 2: Figure S1c). In our dataset, 2542 circRNAs were detected in at least 5 GBC tissues or 5 para-cancer tissues, with significantly higher expression levels.

We found significant differences of circRNA expression patterns between GBC and para-cancer tissues. Only about 20% of circRNA candidates were detected in both cancer and para-cancer tissues (Additional file 2: Figure S1d). For every pair of cancer and para-cancer tissues from the same patient, this number ranged from 14 to 20%, suggesting that the circRNA expression profiles have changed tremendously during cancer initiation and progression (Additional file 2: Figure S1e).

**Fig. 1:**
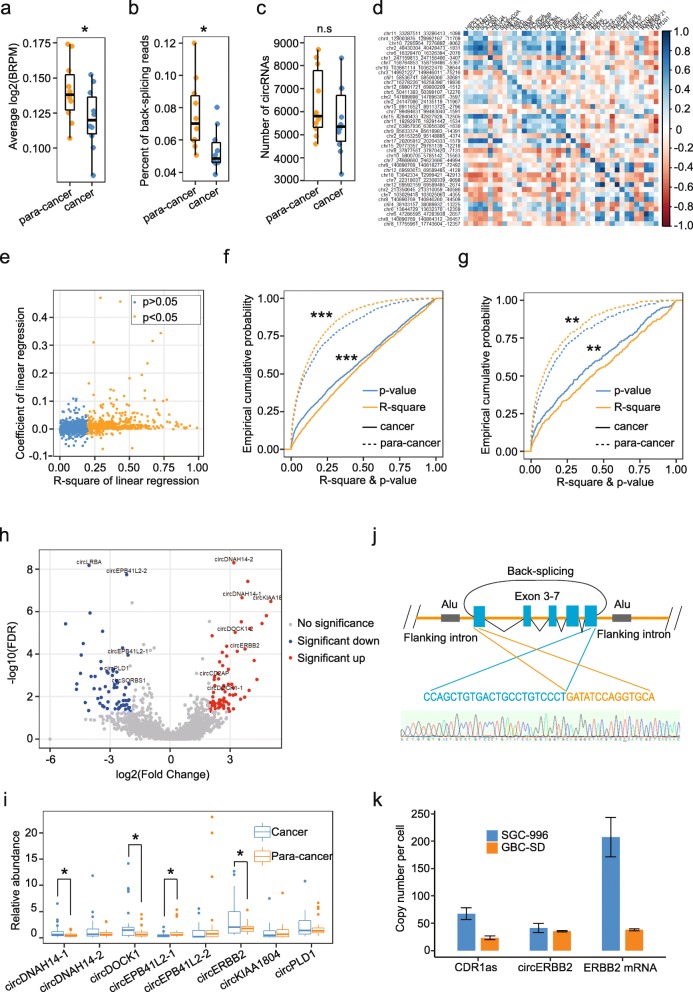
Different circRNA expression patterns between GBC and para-cancer tissues, and circERBB2 is significantly increased in GBC tissues. **a** Average abundance of circRNAs in cancer tissues was significantly lower than para-cancer tissues (*n* = 10). **b** Percentage of back-splicing reads in cancer tissues was significantly lower than para-cancer tissues (*n* = 10). **c** Number of circRNAs identified in cancer tissues was lower than para-cancer tissues but did not reach significance (*n* = 10). **d** Expression of some circRNAs was positively correlated with homogenous mRNA. The color scale indicates the value of Pearson correlation. **e** Among 2482 highly expressed circRNAs, expression of 483 circRNAs were significantly correlated with homogenous mRNA. And most had positive correlation, only 40 had negative correlation. Coefficient, *p*-value of coefficient and R-square were calculated with linear regression. **f** Expression of circRNAs in GBC tissues had significantly increased correlation with homologous mRNAs, compared with para-cancer tissues (*n* = 2482). **g** Expression of circRNAs in GBC tissues had significantly increased correlation with homogenous circRNAs, compared with para-cancer tissues (*n* = 380). **h** Volcano plot showing circRNAs that changed significantly between cancer and para-cancer tissues. **i** Expression of the 8 selected circRNAs was determined by qPCR in 29 pairs of GBC and para-cancer tissues (*n* = 29). **j** Structure and back-splicing sequence of circERBB2. **k** Copy number per cell of circERBB2, CDR1as and ERBB2 mRNA. Quantitative data from three independent experiments were presented as mean ± SD (error bars). *P*-values were determined by paired, two-tailed two sample t-test. FDR value were calculated with BH method. *: *p* < 0.05; **: *p* < 0.01; ***: *p* < 0.001 n.s: not significant

Generally, the expression levels of circRNAs were decreased in GBC tissues, compared with para-cancer tissues. Average BRPM of circRNAs and percentage of back-splicing reads to total reads were both significantly decreased in GBC tissues, (*p* = 0.015 and 0.019) (Fig. 1a, b). And with the same sequencing depth, every cancer tissue was detected with 5610.8 ± 1493.4 kinds of circRNAs, lower than 6397.9 ± 1507.4 in para-cancer tissue, though not reaching the significance (*p* = 0.25) (Fig. 1c).

Back-splicing is regulated by *cis*-elements and *trans*-factors, which are represented by Alu repeats and RNA binding proteins like QKI and ADAR1, respectively [6, 31, 32]. Theoretically, competition between back-splicing and canonical splicing leads to negative correlations between expression of circRNAs and homogeneous mRNAs [33, 34]. However, a set of highly expressed circRNAs in our dataset had obviously positive correlations with homogeneous mRNAs (Fig. 1d). To further clarify regulation of circRNA biogenesis, we analyzed correlations between 2458 highly expressed circRNAs that have homogeneous mRNAs. Among 483 circRNAs that were found to have significant correlations with homogeneous mRNAs, most were positively correlated with their homogeneous mRNAs, but only 40 were negative (Fig. 1e). In addition, the expression levels of circRNAs that generated from the same gene locus tended to have positive correlation with each other (Additional file 2: Figure S1f). It has been reported that some circRNAs show positive correlation with mRNAs as they share same gene locus and transcription regulation [7]. Thus, above data suggest that expression levels of circRNAs are mainly regulated at the transcriptional level, which regulates circRNA expression by altering total amount of pre-mRNA, rather than at the splicing level.

Since we have identified the main way of regulation of circRNA expression, we wondered if some alterations of circRNA regulation happened during GBC development. We found that circRNAs of GBC tissues had a significantly increased correlation with expression of homogeneous mRNAs, compares with para-cancer tissues. We used *p*-value of correlation coefficient and R-square to evaluate the correlation, as increase of correlation is indicated by decrease of *p*-value and increase of R-square. As shown by empirical cumulative probability plots, *p*-value of cancer tissues was significantly decreased while R-square was significantly increased (Fig. 1f). Likewise, the correlation between expression levels of circRNAs from the same gene locus was increased in GBC tissues, compared with para-cancer tissues (Fig. 1g). These results indicate that circRNA regulation is more dependent on modulation of genomic transcription in GBC tissues.

### A circRNA from ERBB2 gene is significantly increased in GBC tissues

Among 2582 highly expressed circRNAs, 131 had fold change> = 4 and FDR < 0.05 (Fig. 1h). Among of them, 62 were significantly increased in cancer tissues, while 69 were significantly decreased (Fig. 1h). With comprehensive consideration of expression level and fold change, we chose 12 circRNAs for further study (Fig. 1h, Table 1). We designed divergent primers (Additional file 1: Table S1) for every circRNA to amplify back-splicing fragment from template of cDNA and genomic DNA (gDNA) extracted from GBC cells. Back-splicing fragments of 9 circRNAs were amplified from cDNA and confirmed by DNA sequencing (Additional file 3: Figure S2a). The putative back-splicing fragment of circSORBS1 was also detected from gDNA, suggesting that circSORBS1 might not be real circRNA (Additional file 3: Figure S2a). Then we performed RNase R resistance assay and found that all these 8 circRNAs were resistant against digestion of RNase R, further confirmed their circular nature (Additional file 3: Figure S2b).

**Table 1:** 12 circRNAs with high expression levels and significant fold changes

circRNA Name	Chromosome	Start site	End site	Homogenous mRNA	Length
circCD2AP	chr6	47,503,279	47,554,766	CD2AP	537
circDNAH14–1	chr1	224,952,669	224,968,874	DNAH14	800
circDNAH14–2	chr1	224,952,669	224,974,153	DNAH14	863
circDOCK1–1	chr10	126,970,701	127,257,429	DOCK1	2801
circDOCK1–2	chr10	126,970,701	127,127,764	DOCK1	2998
circEPB41L2–1	chr6	130,926,604	130,956,499	EPB41L2	719
circEPB41L2–2	chr6	130,955,104	130,956,499	EPB41L2	824
circERBB2	chr17	39,708,320	39,710,481	ERBB2	676
circKIAA1804	chr1	233,346,441	233,362,293	KIAA1804	747
circLRBA	chr4	150,467,672	150,491,035	LRBA	450
circPLD1	chr3	171,642,839	171,692,442	PLD1	1366
circSORBS1	chr10	95,381,684	95,410,777	SORBS1	843

Next we performed qPCR to verify 8 differentially expressed circRNAs in 29 pairs of GBC and para-cancer tissues. Relative abundances of the 8 circRNAs in GBC and para-cancer tissues were generally in line with BRPM values acquired by RNA sequencing. Four circRNAs, which are circDNAH14–1, circDOCK1–1, circEPB41L2–1, and circERBB2, reached significance in qPCR assay (Fig. 1i). We found circERBB2, whose homogeneous mRNA is one of the most important driver gene in GBC [35], changed most obviously. CircERBB2 is transcribed from the ERBB2 gene locus and formed by back-splicing between splicing acceptor of exon 3 and splicing donor of exon 7. It is 676 nt length, containing exon 3, exon 4, exon 5, exon 6, and exon 7 (Fig. 1j). Two flanking introns of circERBB2 both have one and only one reversely aligned Alu repeats with about 90% bases are reversely complementary (Fig. 1j). We assumed that the reverse complementation between two flanking Alu repeats mediated circularization of circERBB2 [36]. The back-splicing sequence was confirmed by DNA sequencing (Fig. 1j). As shown in Fig. 1k, circERBB2 was highly expressed in GBC cell lines SGC-996 and GBC-SD. It had about 40 copies per cells, comparable to CDR1as, which is one of the most abundant circRNAs across many species (Fig. 1k). Robust expression of circERBB2 indicates that it may play essential role in occurrence and progression of GBC.

### CircERBB2 promotes growth of GBC cells

To identify if circERBB2 affects biological behavior of GBC cells, we designed two back-splicing sequence-targeted siRNAs to silence the expression of circERBB2 (Additional file 1: Table S1). Both siRNAs effectively silenced circERBB2 expression with no detectable effects on ERBB2 mRNA (Additional file 4: Figure S3a). We performed CCK8 assay and found that silencing of circERBB2 significantly impaired proliferation of SGC-996 and GBC-SD cells (Additional file 4: Figure S3b, c). In accordance with CCK8 assay, silencing of circERBB2 also impaired clone-formation ability of SGC-996 and GBC-SD cells (Additional file 4: Figure S3d), suggesting that circERBB2 is associated with proliferation of GBC cells.

**Fig. 2:**
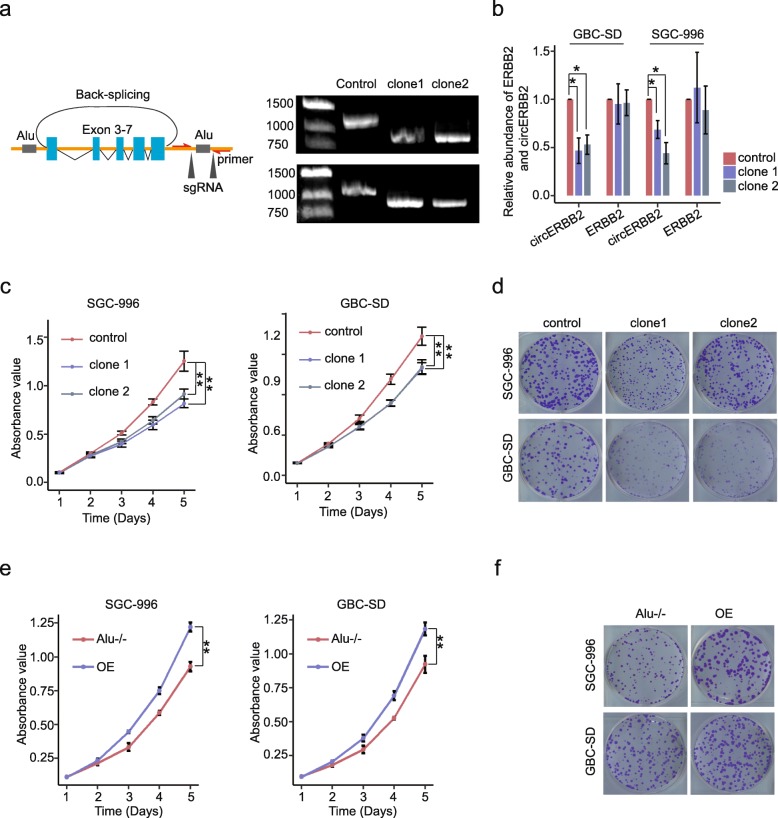
circERBB2 promotes growth of GBC cells in vitro. **a** Deletion of 3′ flanking Alu repeat was achieved with two sgRNAs and verified by PCR using primer pair targeting flanking of sgRNA targeted region. **b** qPCR showed that circERBB2, but not ERBB2 mRNA, was knock-down by deletion of 3′ flanking Alu repeat (*n* = 3). **c** CCK8 assay showed that knock-down of circERBB2 by CRISPR-cas9 impaired proliferation of GBC cells (*n* = 3). **d** Knock-down of circERBB2 by CRISPR-cas9 impaired clone formation ability of GBC cells. **e** OE of circERBB2 promoted proliferation of GBC cells (*n* = 3). **f** OE of circERBB2 promoted clone formation of GBC cells. Quantitative data from three independent experiments was presented as mean ± SD (error bars). *P*-values were determined by paired, two-tailed two sample t-test. *: *p* < 0.05; **: *p* < 0.01

To further provide evidence that circERBB2 is implicated in cancer progression, we designed two sgRNAs that individually target boundaries of the downstream flanking Alu repeat (Additional file 1: Table S1, Fig. 2a). The targeted gDNA region was PCR amplified with primers flanking the targeted gDNA regions, and several homozygous clones of SGC-996 and GBC-SD cells with deleted downstream flanking Alu repeat (SGC-996^Alu−/−^ and GBC-SD^Alu−/−^) were identified by analyzing the length of PCR fragment. Two clones from both cell lines were randomly selected and their targeted gDNA sequence was confirmed by DNA sequencing (Fig. 2a, Additional file 4: Figure S3e). As shown by qPCR assay, circERBB2 in SGC-996^Alu−/−^ and GBC-SD^Alu−/−^ was significantly decreased but still detectable (Fig. 2b). Semi-quantitative PCR with divergent primers followed by agarose gel electrophoresis excluded influence of non-specific product. It suggested that deletion of flanking Alu repeated could inhibit but not eliminate formation of circERBB2. Canonical splicing between exon 7 and exon 8 were not affected and ERBB2 mRNA was also not significantly changed (Fig. 2b). Then we determined proliferation ability of GBC cells by CCK8 and clone-formation assays, showing that proliferation of SGC-996^Alu−/−^ and GBC-SD^Alu−/−^ decreased significantly compared with control cells (*p* < 0.01) (Fig. 2c, d). We also constructed a circERBB2 OE vector and transfected it into SGC-996^Alu−/−^ and GBC-SD^Alu−/−^ cells (Additional file 4: Figure S3f). With transfection of the circERBB2 expression vector, ectopic expression of circERBB2 increased 50–100 folds (Additional file 4: Figure S3f). By employing the CCK8 and clone formation assays, we found that OE of circERBB2 recovered the proliferation ability of GBC cells (*p* < 0.01) (Fig. 2e-f). Thus, these results validate that circERBB2 is associated with proliferation of GBC cells.

**Fig. 3:**
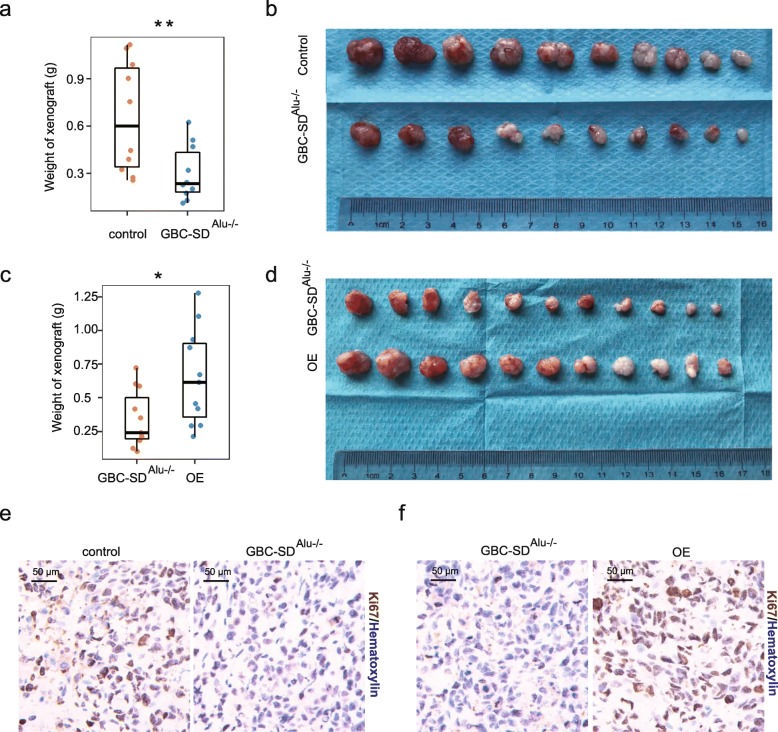
circERBB2 promotes growth of GBC cells in vivo. **a** Weight of xenografts decreased significantly in GBC-SD^Alu−/−^ cells, compared with GBC-SD cells (*n* = 10). **b** Xenograft size of GBC-SD^Alu−/−^ was significantly smaller than GBC-SD cells. **c** Weight of xenografts increased significantly in GBC-SD^Alu−/−^ cells with OE of circERBB2, compared with GBC-SD^Alu−/−^ cells without OE of circERBB2 (*n* = 11). **d** Xenograft size of cells with OE of circERBB2 was significantly larger than GBC-SD^Alu−/−^ cells. **e** Proportion of Ki67(+) cells decreased significantly in GBC-SD^Alu−/−^ xenografts. **f** Proportion of Ki67(+) cells increased significantly in xenografts with OE of circERBB2. Quantitative data was presented as mean ± SD (error bars). *P*-values were determined by unpaired, two-tailed two sample t-test. *: *p* < 0.05; **: *p* < 0.01

Next, to see if circERBB2 affects in vivo proliferation of GBC cells, we injected control GBC-SD and GBC-SD^Alu−/−^ cells into subcutaneous tissue of nude mouse. We observed that the weight of xenografts decreased significantly in GBC-SD^Alu−/−^ cells, compared with control cells (*p* < 0.01) (Fig. 3a), and xenograft tumors of GBC-SD^Alu−/−^ were obviously smaller than those of control cells (Fig. 3b). Moreover, in vivo tumor growth of GBC-SD^Alu−/−^ cells was remarkably promoted by OE of circERBB2 (Fig. 3c). Xenograft tumors from GBC-SD^Alu−/−^ that stably transfected with circERBB2 OE vector were significantly larger than control group (Fig. 3d). Moreover, percentage of Ki67(+) cells was significantly less in GBC-SD^Alu−/−^ cells, compared with control cells (Fig. 3e), whereas OE of circERBB2 significantly increased percentage of Ki67(+) cells (Fig. 3f). Collectively, the above results confirm that circERBB2 promotes growth of GBC cells, both in vitro and in vivo.

### CircERBB2 promotes rDNA transcription and pre-rRNA (45S) synthesis in the nucleolus

Since most circRNAs are reported to work as miRNA sponge in cancer [13–15], we analyzed potential miRNA binding sites in circERBB2 with CircInteractome, a web tool for exploring circRNAs and their interacting miRNAs [37]. CircERBB2 was predicted to bind with several miRNAs (Additional file 5: Figure S4a). However, unlike CDR1as that has dozens of binding sites targeting one specific miRNA (miR-7) [5], all potential miRNA targets of circERBB2 have only one or two binding sites (Additional file 5: Figure S4a) and no obvious functional similarity or relation between those miRNAs was found. We performed gene ontology (GO) analysis of genes targeted by those miRNAs, but the result was unrelated with cellular proliferation (Additional file 5: Figure S4b). We highly doubted that circERBB2 might work through unknown mechanisms beyond miRNA sponge.

**Fig. 4:**
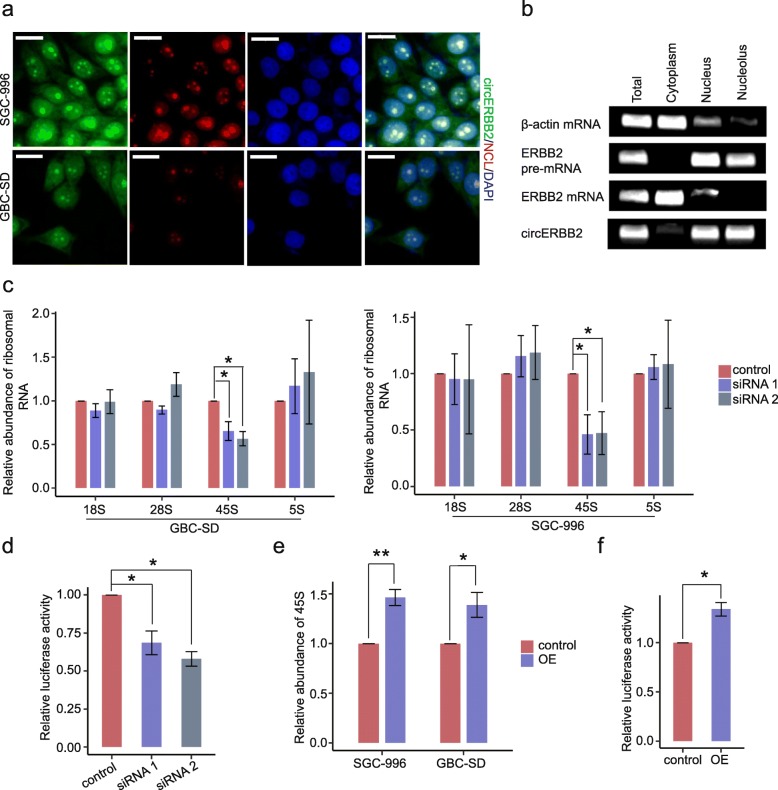
circERBB2 promotes rDNA transcription in the nucleolus. **a** FISH + IF double staining showed nucleolar localization of circERBB2. Scale bar: 20 μm. **b** circERBB2 accumulated to the nucleoli. Total RNA from SGC-996 cells was separated into cytoplasmic, nuclear, and nucleolar fractions and analyzed by semi-quantitative PCR. **c** qPCR determined relative abundance of rRNAs after silencing of circERBB2, and steady level of 45S pre-rRNA decreased significantly (*n* = 3). **d** Luciferase reporter assay showed that silencing of circERBB2 decreased rDNA promoter activity (*n* = 3). **e** qPCR assay showed that relative steady level of 45S increased significantly with OE of circERBB2 (*n* = 3). **f** Luciferase reporter assay showed that OE of circERBB2 increased rDNA promoter activity (*n* = 3). Quantitative data from three independent experiments was presented as mean ± SD (error bars). *P*-values were determined by paired, two-tailed two sample t-test. *: *p* < 0.05; **: *p* < 0.01

The function of non-coding RNA is closely associated with its subcellular location pattern [38]. To get insight into the mechanism of how circERBB2 promotes GBC proliferation, we performed FISH with a FITC-labeled RNA probe targeting back-splicing sequence (Additional file 1: Table S1, Additional file 5: Figure S4c). Unlike canonical circRNAs that are localized in cytoplasm and work as miRNA sponges, circERBB2 was enriched in some area of the nucleus where resemble nucleoli, both in SGC-996 and GBC-SD cells (Additional file 5: Figure S4d). We performed co-staining of circERBB2 and the nucleolar marker nucleolin (NCL) and confirmed obvious co-localization between circERBB2 and NCL in the nucleoli, although weaker florescent signal of circERBB2 was also detected in the cytoplasm and the nucleoplasm (Fig. 4a). Subcellular fraction followed by semi-quantitative PCR further validated nucleolar enrichment of circERBB2, as circERBB2 showed similar sub-cellular localization with ERBB2 pre-mRNA (Fig. 4b).

Ribosome synthesis, which is mainly happened in the nucleolus, is the rate-limiting step of cellular proliferation [20]. Abnormally rapid synthesis of rRNA is necessitated by malignant proliferation of cancer cells [20]. As circERBB2 was accumulated in the nucleolus, we speculated whether circERBB2 promoted GBC cells progression by regulating rRNA synthesis. We tested abundances of 45S, 18S, 28S, and 5S rRNA in GBC cells with or without silencing of circERBB2. As shown by qPCR assay, while no detectable change of 18S, 28S, and 5S was found, steady-state level of 45S, the direct product of Pol I transcription, decreased significantly after silencing of circERBB2, indicating that circERBB2 was associated with transcription of rDNA (Fig. 4c). Then to verify this, we performed a dual-luciferase report assay. The result showed that the rDNA promoter activity was markedly decreased after silencing of circERBB2 (Fig. 4d). Moreover, steady level of 45S and rDNA promoter activity were obviously increased in cells with OE of circERBB2 (Fig. 4e, f). Taken together, the results demonstrate that circERBB2 promotes GBC proliferation by up-regulating the Pol I activity and rDNA transcription in the nucleoli.

### CircERBB2 promotes rDNA transcription by interacting with PA2G4

**Fig. 5:**
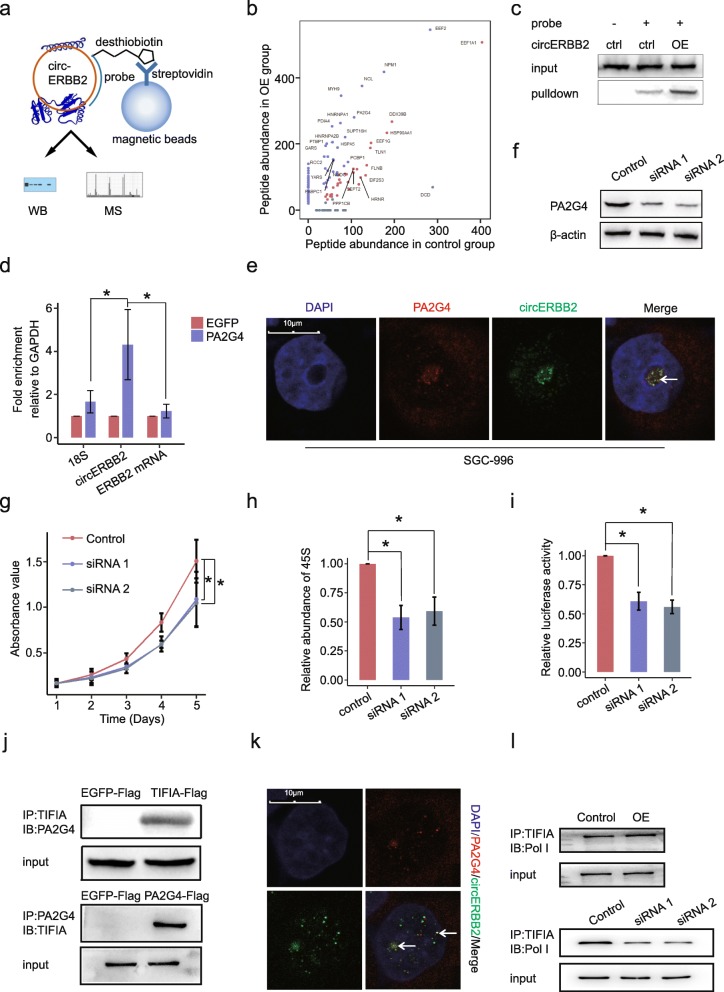
circERBB2 interacts with PA2G4. **a** Schematic plot of circRNA pulldown assay with a desthiobiotin-labeled DNA probe. **b** Potential circERBB2-associated proteins identified by mass spectrum. Proteins in purple, which had significant higher abundance in OE group than in control group, were protein candidates that specifically interacted with circERBB2. **c** RNA pulldown followed by western blot confirmed interaction between circERBB2 and PA2G4. **d** RIP followed by qPCR confirmed interaction between circERBB2 and PA2G4. 18S and ERBB2 mRNA were used as negative control (*n* = 3). **e** FISH+IF double staining showed co-localization of circERBB2 (green) and PA2G4 (red) in the nucleolus. Co-localization was indicated by arrowhead. Scale bar, 10 μm. **f** PA2G4 was effectively silenced by 2 siRNAs. β-actin was used as internal control. **g** CCK8 assay showed that silencing of PA2G4 impaired proliferation of SGC-996 cells (*n* = 3). **h** qPCR assay showed that silencing of PA2G4 decreased steady level of 45S pre-rRNA (*n* = 3). **i** Silencing of PA2G4 decreased rDNA promoter activity (*n* = 3). **j** Co-IP followed by western blot confirmed interaction between PA2G4 and TIFIA. **k** IF double staining showed co-localization of TIFIA (green) and PA2G4 (red) in the nucleolus. Co-localization was indicated by arrowhead. Scale bar, 10 μm. **l** OE of circERBB2 significantly increased interaction between TIFIA and Pol I, and silencing of circERBB2 decreased interaction between TIFIA and Pol I. Quantitative data from three independent experiments was presented as mean ± SD (error bars). *P*-values were determined by paired, two-tailed two sample t-test. *: *p* < 0.05; **: *p* < 0.01

Next, we asked how circERBB2 promoted rDNA transcription in GBC cells. We designed a desthiobiotin-labeled DNA probe and performed RNA pulldown assay to identify circERBB2-associated proteins (Fig. 5a). 293 T cells were transfected with control or circERBB2 OE vector, respectively, and total RNA from the two groups was incubated with the DNA probe to capture circERBB2. Through qPCR assay, we confirmed that the DNA probe could effectively and specifically capture circERBB2. The DNA probe captured obviously more circERBB2 when incubated with total RNA with OE of circERBB2 (Additional file 6: Figure S5a).

Proteins interact with circERBB2 would be significantly enriched in RNA pulldown assay and their abundances would be higher in OE group. By mass spectrometric analysis, we identified dozens of proteins in both groups. Among proteins enriched in OE group were some nucleolus-associated proteins like NCL, NPM1, and HNRNPA1, further confirmed that circERBB2 was associated nucleolar function (Fig. 5b). In contrast, some of the canonical RNA binding proteins like DDX39B and EEF1A1, and some of the canonical protein chaperones like HSP90AA1, were enriched in both groups, indicating that they were non-specifically bound to diverse RNA molecules or streptavidin beads (Fig. 5b). Besides, AGO2, an indicator of circRNA-miRNA interaction, was not found in both groups.

Among nucleolus-associated proteins that enriched in OE group, a specific one was PA2G4, which was reported to modulate rDNA transcription, rRNA processing and cellular proliferation [39, 40]. RNA pulldown followed by western blot confirmed the interaction between circERBB2 and PA2G4 (Fig. 5c). The RIP assay also showed that circERBB2 was significantly enriched in RNA binding to Flag-tagged PA2G4, compared with the control Flag-tagged EGFP (Fig. 5d). Through FISH+IF dual staining assay, we found both circERBB2 and PA2G4 were enriched in the nucleoli (Additional file 6: Figure S5b). Then we used a confocal microscope and observed obvious co-localization between them in the nucleoli (Fig. 5e). All of these results confirmed that circERBB2 interacts with PA2G4 in the nucleoli.

PA2G4 has two functional distinct isoforms, which are p42 and p48 [41]. While p42, the unstable and minor isoform, works as a tumor suppressor by inhibiting cancer growth and promoting cellular differentiation, p48 is the main isoform that works as an oncogene by promoting cancer cell proliferation [41]. Through analysis of PA2G4 abundances in 28 pairs of GBC and para-cancer tissues by western blot, we found that p48 isoform increased remarkably in GBC tissues, while p42 was usually undetectable (Additional file 6: Figure S5c, d). Simultaneously, PA2G4 mRNA increased significantly in GBC tissues (Fig. 5e). These results suggest that PA2G4 may work as an oncogene in GBC.

Then we silenced PA2G4 expression by two siRNAs in SGC-996 cells (Fig. 5f), and found that silencing of PA2G4 impaired proliferation of SGC-996 cells (Fig. 5g). The qPCR and dual-luciferase assays indicated that silencing of PA2G4 decreased steady level of 45S and promoter activity of rDNA, confirming that PA2G4 is essential for rDNA transcription, as like circERBB2 (Fig. 5h, i).

In T-cells, PA2G4 has been reported to regulate rDNA transcription by interacting with TIFIA, a key regulator of Pol I activity and rDNA transcription [39, 40]. As in T-cells, we confirmed that PA2G4 directly bound to TIFIA in GBC cells (Fig. 5j) and obvious co-localization between TIFIA and PA2G4 was observed in the nucleoli (Fig. 5k), suggesting that PA2G4 is an important regulator of TIFIA activity in GBC.

TIFIA is an essential co-factor of rDNA transcription that directly bind to Pol I [39]. And we found that silencing of TIFIA severely decreased abundances of rRNAs and rDNA promoter activity in GBC cells (Additional file 6: Figure S5f). As circERBB2 directly bound to PA2G4, which is an important regulator of TIFIA, we performed co-IP to see if circERBB2 affects the interaction between TIFIA and Pol I. We found that the interaction between Pol I and TIFIA was significantly increased by OE of circERBB2, and decreased by circERBB2 knock-down (Fig. 5l) Thus, we confirmed that circERBB2 and PA2G4 interacted with each other in the nucleoli, and they had similar function in promoting GBC cell proliferation and rDNA transcription. These findings demonstrate that a circERBB2-PA2G4-TIFIA regulatory axis plays an essential role in up-regulating Pol I activity, rDNA transcription, and cellular proliferation in GBC cells.

### CircERBB2 regulates nucleolar localization of PA2G4

**Fig. 6:**
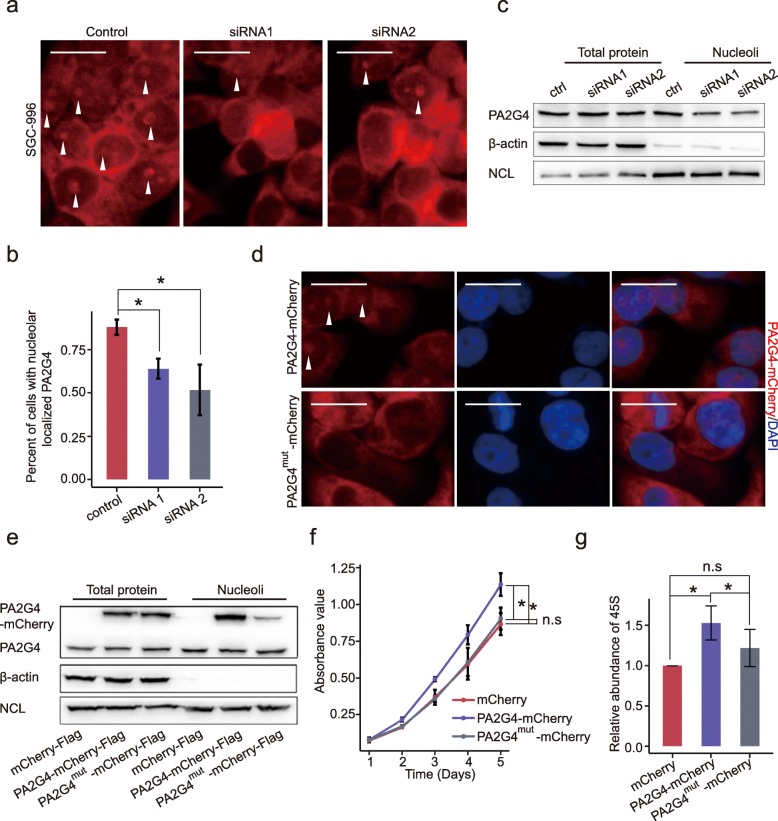
circERBB2 regulates nucleolar-localization of PA2G4. **a** IF assay showed that silencing of circERBB2 decreased nucleolar localization of PA2G4. Arrowhead showed nucleoli stained with PA2G4. Scale bar: 20 μm. **b** Quantitative analysis of the percentage of cells with nucleolar enrichment of PA2G4 (*n* = 4). **c** Western blot showed that silencing of circERBB2 had no effect on total amount PA2G4, but significantly decreased nucleolar localized PA2G4. β-actin and NCL were used as internal control for total and nucleolar protein, respectively. **d** IF showed that mutation of polybasic region impaired nucleolar enrichment of PA2G4. Arrowhead showed nucleoli stained with PA2G4. Scale bar: 20 μm. **e** Western blot showed that mutation of polybasic region impaired nucleolar enrichment of PA2G4. Distribution of endogenous PA2G4 was unaffected. **f** CCK8 assay showed that PA2G4-mCherry, but not PA2G4^mut^-mCherry, effectively promoted proliferation of SGC-996 cells (*n* = 3). **g** PA2G4-mCherry, but not PA2G4^mut^-mCherry, promoted rDNA transcription in GBC cells (*n* = 3). Quantitative data from three independent experiments was presented as mean ± SD (error bars). *P*-values were determined by paired, two-tailed two sample t-test. *: *p* < 0.05; n.s: not significant

Next, we asked how circERBB2 affects the function of PA2G4. No detectable change of total amount of PA2G4 was found after silencing of circERBB2. However, through IF assay, we found that the nucleolar localization of PA2G4 decreased obviously when circERBB2 was silenced (Fig. 6a). While SGC-996 cells treated with scramble had 88.1 ± 4.4% cells showed obvious nucleolar enrichment of PA2G4, this number decreased to only 64 ± 5.7% and 51.7 ± 14.7% of cells treated with siRNA 1 and siRNA 2, respectively (Fig. 6b). We also performed the nucleoli isolation and further determined change of PA2G4 in the nucleoli by western blot, showing that total amount of PA2G4 was not changed while PA2G4 in the nucleoli was significantly decreased (Fig. 6c). These results confirm that circERBB2 regulates the nucleolar localization of PA2G4.

Subcellular localization of PA2G4 is an intriguing problem needs further clarification. Through IF assay, we found that when SGC-996 cells were cultured in FBS-free medium, nucleolar localized PA2G4 decreased significantly (Additional file 7: Figure S6a), indicated that subcellular localization of PA2G4 was associated with cellular proliferative states. Since the transcription of rDNA is happened in the nucleolus, we assumed that nucleolar localization was essential for PA2G4 to regulate rDNA transcription and cellular proliferation. So we used NoD, a web server, to predict the nucleolar localization sequence of PA2G4 [42]. A C-terminal polybasic region that consists of seven lysines and an arginine was predicted to play a critical role in nucleolar translocation of PA2G4 (Additional file 7: Figure S6b). We constructed a mCherry-Flag-tagged PA2G4 expression vector (PA2G4-mCherry-Flag) and then the 364–373 amino acids were mutated from N-RKTQKKKKKK-C to N-RATQAAAAAA-C (PA2G4^mut^-mCherry-Flag). As shown by IF and western blot assays, wild type PA2G4 was enriched in the nucleoli while PA2G4^mut^ was mainly localized in the cytoplasm (Fig. 6d, e).

Then, we tested if the translocation of PA2G4 from nucleoli has any influence on proliferation of GBC cells. SGC-996 cells were pre-treated with siRNA to decrease endogenous PA2G4 protein and then transfected with the control vector mCherry-Flag, PA2G4-mCherry-Flag, or PA2G4^mut^-mCherry-Flag plasmid, respectively. PA2G4-mCherry-Flag significantly promoted proliferation of SGC-996 compared to that of the control vector, whereas there was no significant difference between mCherry-Flag and PA2G4mut-mCherry-Flag (Fig. 6f). Meanwhile, PA2G4-mCherry-Flag but not PA2G4^mut^-mCherry-Flag, significantly increased the steady level of 45S (Fig. 6g), indicating that nucleolar localization was essential for PA2G4 to promote rDNA transcription and GBC cellular proliferation. Thus, above results suggest that the function of circERBB2 is, at least partly, mediated by regulating the nucleolar localization of PA2G4.

### High expression of circERBB2 indicates worse prognosis of GBC patients

**Fig. 7:**
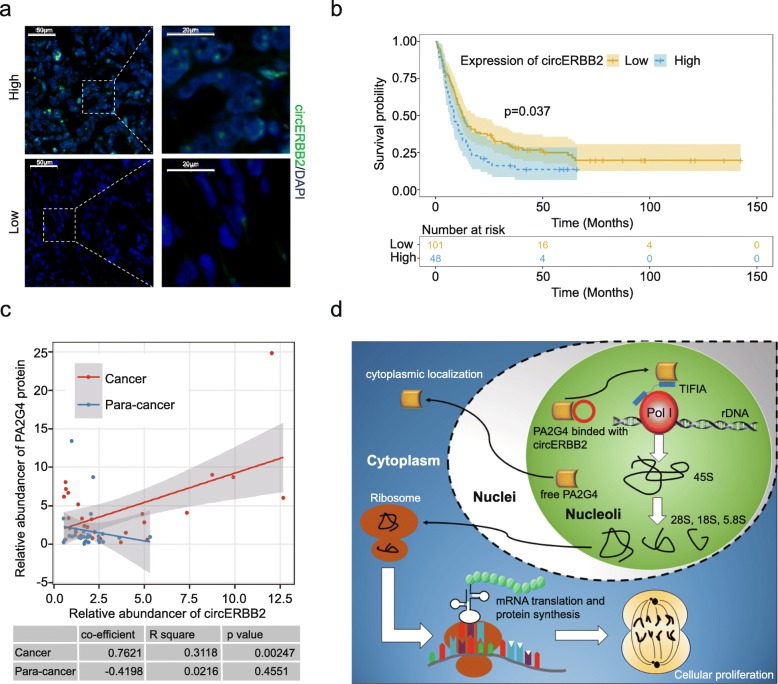
Increased expression of circERBB2 indicates worse prognosis of GBC patients. **a** CircERBB2 expression level was determined by FISH. **b** Survival probability was calculated with Kaplan-Meier survival analysis. Increased expression of circERBB2 indicated worse prognosis of GBC patients (*n* = 149). **c** circERBB2 showed significantly positive correlation with PA2G4 in GBC tissues, but not in para-cancer tissues (*n* = 28). **d** Proposed model of the circERBB2-PA2G4-TIFIA complex in upregulating rDNA transcription and GBC progression

To evaluate clinical significance of circERBB2 with respect to prognosis of GBC patients, FISH assay was performed with a tissue microarray containing 149 surgically removed primary GBC specimens. The expression level of circERBB2 was higher in 48 patients and relatively lower in other 101 patients (Fig. 7a). We compared overall survival (OS) of the two groups and found that GBC patients with high circERBB2 expression had significantly shorter OS time (Fig. 7b). Median survival times were 12.73 months and 8.54 months for the circERBB2-high and -low groups, respectively (*p* = 0.037). Patients of the two groups had approximately equal age and sex distribution.

Next we evaluated the correlation between circERBB2 expression level and clinicopathological status that were tumor size, T stage, lymph metastasis, and liver metastasis. Percent of patients with tumor size > 3 cm, T-stage > = 3, lymph metastasis and liver metastasis were all higher in the group with higher circERBB2 expression (Table 2). However, it may be due to the limited patient number or other influence factors, no significance was reached for all the four clinicopathological status.

**Table 2:** correlation between clinicopathological status and expression of circERBB2

Clinicopathological status	circERBB2 expression		*P*-value
	Low	High	
Tumor size			
< 3 cm	48(47.5%)	20 (41.6%)	0.6207
> 3 cm	53 (52.5%)	28 (58.4%)	
T-stage			
T1–2	21 (20.8%)	5 (10.4)	0.1841
T3–4	80 (79.2%)	43 (89.6%)	
Lymph metastasis			
No	65 (64.4%)	28 (58.4%)	0.5972
Yes	36 (35.6%)	20 (41.6%)	
Liver metastasis			
No	65 (64.4%)	27 (56.2%)	0.4407
Yes	36 (35.6%)	21 (43.8%)	

Interestingly, we found that in GBC tissues, but not para-cancer tissues, the expression level of circERBB2 was positively correlated with abundance of PA2G4 protein (Fig. 7c), suggesting that circERBB2 promoted GBC progression in synergy with PA2G4. Our data illuminates that circERBB2 regulates nucleolar localization of PA2G4, thus forming a circERBB2-PA2G4-TIFIA regulatory axis to up-regulate Pol I activity, rDNA transcription, and GBC progression (Fig. 7d). CircERBB2 has the potential to be a new therapeutic target of GBC.

## Discussion

The present study profiled circRNA expression pattern in GBC and para-cancer tissues, and found dozens of circRNAs that changed significantly during GBC progression. CircERBB2, a circRNA from the ERBB2 gene locus that increased remarkably in GBC tissues, was found to promote GBC progression by regulating PA2G4-dependent rDNA transcription and cellular proliferation. And the circERBB2 expression level was associated with prognosis of GBC patients.

In many cancer types, circRNA expression patterns change tremendously during cancer progression [17]. In GBC tissues, a remarkable feature of circRNA transcripts is that total abundances of circRNA decrease significantly, compared with para-cancer tissues. This is in accordance with previous studies that total circRNA abundances were decreased in prostate cancer and glioma [9, 17]. As we know, circRNAs tend to accumulate in proliferation-inactive cells or senescent cells [43]. Compared with GBC tissue that is under malignant mitosis, para-cancer tissue is proliferation-inactive and more differentiated. We think that active mitosis in cancer tissue leads to dilution of circRNAs in cells, as previous reported in prostate cancer [17]. If so, the expression alterations of some circRNAs that are decreased in cancer tissues may be caused by passive dilution of total circRNA abundance. Then these circRNAs are unlikely to affect cancer initiation and progression, compared with circRNAs that are increased in cancer tissues. When screening for circRNAs that are associated with cancer biology, more attention shall be paid to genes that are increased in cancer tissues.

Most circRNAs were reported to work as miRNA sponge. Like most reported circRNAs, circERBB2 has dozens of potential miRNA binding sites targeting different miRNAs, but these miRNAs seem functional unrelated and most of them have no relation with cellular proliferation. More importantly, unlike previously reported circRNAs that are localized in cytoplasm, circERBB2 is enriched in nucleoli. The function of circRNAs shall be associated with their subcellular location patterns [38]. We confirm that circERBB2 is associated with transcription of rDNA, which is happened in nucleoli and control the rate of cellular proliferation [20]. Our work indicates that circRNAs have diverse functions, far more than only being miRNA sponges. It is necessary and also helpful to determine the subcellular localization of circRNAs before affirming that they work as miRNA sponges or others.

PA2G4 is an evolutional conserved protein with diverse functions involving cellular proliferation, differentiation, signaling transduction and rRNA processing [39–41]. It has two functional distinctive isoforms, which are p42 and p48. In our work, p42 is almost undetectable in most GBC tissues, while p48 is increased remarkably in GBC and works as an oncogene by upregulating Pol I activity and rDNA transcription. PA2G4 is closely associated with nucleolar function, but unlike canonical nucleolar related proteins, it has no decisive nucleolar localization peptide [36]. The C-terminal poly basic region identified in our work plays essential role but still, it is ancillary and cannot entirely determine the subcellular fate of PA2G4. Given its indispensable cellular function, the subcellular fate of PA2G4 would be tightly regulated by various factors. Our work identified circERBB2 as one of the critical regulators, although a more detailed mechanism is still lacking. As circERBB2 has high expression level (Fig. 1k) and it is associated with several abundant nucleolar proteins like NCL and NPM1 (Fig. 5b), we speculate that circERBB2 may work as RNA scaffold to anchor some proteins from escaping from the nucleoli. But we need further study to be conclusive.

For all we know, circERBB2 is the first circRNA that is reported to regulate progression of cancer by promoting rDNA transcription. Mechanisms of how circRNAs work are far more complicated than expectation. Our work indicates that subcellular fates of circRNAs are diverse and are closely related with their functions. CircRNAs have tremendous regulatory potential and play huge roles in cancer initiation and progression, and their significance in cancer therapy merits further study.

## Conclusions

Our study demonstrates that circRNA expression pattern changed tremendously during GBC development, and circERBB2 is increased significantly in GBC tissues. CircERBB2, which accumulates in nucleoli and regulates rDNA transcription, promotes GBC proliferation, in vitro and in vivo. CircERBB2 regulates nucleolar localization of PA2G4, thereby forming a circERBB2-PA2G4-TIFIA regulatory axis to modulate Pol I activity, rDNA transcription, and GBC growth. Increased expression of circERBB2 is associated with worse prognosis of GBC patients. CircERBB2 serves as an important regulator of cancer cell proliferation, and it shows the potential to be a new therapeutic target of GBC.

## Supplementary information

**Additional file 1: Table S1.** Sequence of Oligonucleotide. **Table S2.** Critical Reagent.

**Additional file 2: Figure S1.** Different circRNA expression patterns between GBC and para-cancer tissues. **a** Expression levels of different circRNAs varied tremendously, BRPM values of different circRNAs ranged from less than 0.1 to more than 100. **b** Most circRNAs expressed at very low level, with more than 95% circRNAs had BRPM< 1. **c** circRNAs that detected in more samples tended to have higher expression level. **d** Only about 20% of circRNAs were detected in both cancer and para-cancer tissues. **e** For each patient, only 14 to 20% circRNAs were found in both cancer and para-cancer tissues. **f** circRNAs that generated from same gene locus tended to had positive correlation with each other. The color scale indicates the value of Pearson correlation.

**Additional file 3: Figure S2.** Screen for functional circRNAs in GBC tissues. **a** PCR validation of 12 circRNAs with divergent primers. cDNA or gDNA from SGC-996 cells were used as template. Arrows showed bands detected with back-splicing sequence by DNA sequencing. **b** Relative abundance of the 8 circRNAs increased significantly after treatment of RNase R (*n* = 3). Quantitative data from three independent experiments was presented as mean ± SD (error bars). *P*-values were determined by paired, two-tailed two sample t-test. *: *p* < 0.05.

**Additional file 4: Figure S3.** circERBB2 promotes growth of GBC cells in vitro. **a** qPCR showed that circERBB2, but not ERBB2 mRNA, was effectively silenced by two siRNAs targeting back-splicing sequence of circERBB2 (*n* = 3). **b** CCK8 assay showed that silencing of circERBB2 by siRNAs impaired proliferation of SGC-996 cells (*n* = 3). **c** CCK8 assay showed that silencing of circERBB2 by siRNAs impaired proliferation of GBC-SD cells (*n* = 3). **d** Silencing of circERBB2 by siRNAs impaired clone formation ability of GBC cells. **e** gDNA sequence of sgRNA-targeted region of SGC-996^Alu−/−^ and GBC-SD^Alu−/−^ cells. **f** Structure of pLenti-CMV-circERBB2 vector and relative abundance of circERBB2 in GBC cells with or without OE of circERBB2 (*n* = 3). Quantitative data from three independent experiments was presented as mean ± SD (error bars). *P*-values were determined by paired, two-tailed two sample t-test. *: *p* < 0.05; **:*p* < 0.01.

**Additional file 5: Figure S4.** Nucleolar localization of circERBB2. **a** Schematic plot showed miRNAs that predicted as targets of circERBB2 by using circular RNA Interactome. **b** GO analysis of genes targeted by those miRNAs, and the results were unrelated with cellular proliferation. **c** Schematic plot of FISH assay with biotin-label RNA probe targeting back-splicing sequence of circERBB2. **d** FISH assay revealed sub-cellular localization of circERBB2. Scale bar: 20 μm.

**Additional file 6: Figure S5.** circERBB2 interacts with PA2G4. **a** qPCR showed that desthiobiotin-labeled DNA probe effectively captured circERBB2 (*n* = 3). **b** FISH+IF double staining showed that both circERBB2 and PA2G4 was accumulated in the nucleolus. Scale bar, 5 mm. **c, d** Western blot showed that PA2G4 protein increased significantly in GBC tissue, compared with para-cancer tissues (*n* = 28). **e** PA2G4 mRNA increased significantly in GBC tissues, compared with para-cancer tissues (*n* = 29). **f** qPCR showed silencing of TIFIA with two siRNAs severely impaired rDNA transcription and rRNA genesis in GBC cells (*n* = 3). Quantitative data from three independent experiments was presented as mean ± SD (error bars). *P*-values were determined by paired, two-tailed two sample t-test. *:*p* < 0.05; **:*p* < 0.01.

**Additional file 7: Figure S6.** circERBB2 regulates nucleolar-localization of PA2G4. **a** IF showed nucleolar localization of PA2G4 was decreased when SGC-996 cells were cultured in FBS-free medium. **b** Screen for nucleolar localization sequence of PA2G4 with NoD.

## Data Availability

Please contact the corresponding author for all data requests.

## Abbreviations

BRPM: Back-splicing reads per million reads
CCK8: Cell counting kit-8
circRNA: Circular RNA
FISH: Fluorescence in situ hybridization
GBC: Gallbladder cancer
gDNA: Genomic DNA
IF: Immunofluorescence
IHC: Immunohistochemistry
NCL: Nucleolin
OE: Over-expression
OS: Overall survival
Pol I: RNA polymerase I
pre-mRNA: Precursor mRNAs
rDNA: Ribosomal DNA
RIP: RNA-protein immunoprecipitation
rRNA: Ribosomal RNA
rt-PCR: Reverse transcription PCR
RVC: Ribonucleoside Vanadyl Complexes
