# Correction to: Circular RNA circERBB2 promotes gallbladder cancer progression by regulating PA2G4-dependent rDNA transcription

**DOI:** 10.1186/s12943-022-01563-3

**Published:** 2022-06-02

**Authors:** Xince Huang, Min He, Shuai Huang, Ruirong Lin, Ming Zhan, Dong Yang, Hui Shen, Sunwang Xu, Wei Cheng, Jianxiu Yu, Zilong Qiu, Jian Wang

**Affiliations:** 1 Department of Biliary-Pancreatic Surgery, Renji Hospital, School of Medicine, Shanghai Jiao Tong University, 160 Pujian Road, Shanghai, 200127 People’s Republic of China; 2 Basic Research Laboratory, Department of Biliary-Pancreatic Surgery, Renji Hospital, School of Medicine, Shanghai Jiao Tong University, 160 Pujian Road, Shanghai, 200127 People’s Republic of China; 3 Department of Biochemistry and Molecular Cell Biology, Shanghai Key Laboratory of Tumor Microenvironment and Inflammation, Shanghai Jiao Tong University School of Medicine, Shanghai, 200025 China; 4 Institute of Neuroscience, State Key Laboratory of Neuroscience, CAS Center for Excellence in Brain Science and Intelligence Technology, University of Chinese Academy of Sciences, Chinese Academy of Sciences, Shanghai, 200031 People’s Republic of China

**Correction to: Mol Cancer 18, 166 (2019)**

**https://doi.org/10.1186/s12943-019-1098-8**

Following the publication of the article [1], the authors identified an error in the author name of Min He, which was originally presented as ‘Ming He’. The corrected author list can be found here.

The original article has been corrected.
