# Supplementary material for: Circular RNA circERBB2 promotes gallbladder cancer progression by regulating PA2G4-dependent rDNA transcription
Source: Mol Cancer. 2019 Nov 21;18:166. doi: 10.1186/s12943-019-1098-8 (PMC6868820; doi:10.1186/s12943-019-1098-8)
Supplement: Supplementary file 1 — Additional file 1: Table S1. Sequence of Oligonucleotide. Table S2. Critical Reagent. [file 12943_2019_1098_MOESM1_ESM.docx]

**Table S1: sequence of oligonucleotide**

| Primers | |
| --- | --- |
| Primer name | sequence |
| circCD2AP-F | ACAAGTTGGGACTGTTTCCC |
| circCD2AP-R | CCCTTCCTCCTGTAGCTTTTTC |
| circDNAH14-1-F | ACCTACTTTGGAATGGCTATCAGA |
| circDNAH14-1-R | TCTTGGTTTCCTCCTTGTCCA |
| circDNAH14-2-F | TGGCTATCAGAAAGAAGACATTACT |
| circDNAH14-2-R | TGGTTTCCTCCTTGTCCATCTC |
| circDOCK1-1-F | GTGAACCGAACCGTCATTTCC |
| circDOCK1-1-R | GTAACCTCGGTACCACCCTTC |
| circDOCK1-2-F | AACGTTTACCCCTTCGACTG |
| circDOCK1-2-R | TGTAACCTCGGTACCACCCT |
| circEPB41L2-1-F | ACCAAGGAAGTGCAGACCAA |
| circEPB41L2-1-R | TCTTCACTTCAGACACAGAGCC |
| circEPB41L2-2-F | TGACCTGGAGAAACATGCCAA |
| circEPB41L2-2-R | TTCACTTCAGACACAGAGCCT |
| circKIAA1804-F | CAGCTAAACCAGGAGAAGCCC |
| circKIAA1804-R | CATTCCCTCGCCAACCCAAA |
| circLRBA-F | TGCCCACCAACTTCAGAGA |
| circLRBA-R | ATCACAGCAACTCTGTTTGCC |
| circPLD1-F | CGGCGGAGGAAATGCTCTAC |
| circPLD1-R | CACATGATCCGGGTGTCTCA |
| circSORBS1-F | GCAGAGCCCAAGAGCATTTAC |
| circSORBS1-R | ACTGATGGAAAGGCTTGGGAA |
| circERBB2-F | ACGTTTGAGTCCATGCCCAA |
| circERBB2-R | TTGTGAGCGATGAGCACGTA |
| ERBB2-mRNA-F | CGGAAGTACACGATGCGGAG |
| ERBB2-mRNA-R | AAAAGCGCCAGATCCAAGCA |
| GAPDH-F | CTCTCTGCTCCTCCTGTTCGACAG |
| GAPDH-R | AGGGGTCTTACTCCTTGGAGGCCA |
| PA2G4-F | CGGTACTGAGCCTGTGTGAG |
| PA2G4-R | CTGGTCGCTCTTCAAAGGGG |
| 45S-F | TGTCAGGCGTTCTCGTCTC |
| 45S-R | AGCACGACGTCACCACATC |
| 5S-F | CCATACCACCCTGAACGCGC |
| 5S-R | AGCACCCGGTATTCCCAGGC |
| 28S-F | AGAGGTAAACGGGTGGGGTC |
| 28S-R | GGGGTCGGGAGGAACGG |
| 18S-F | GATGGTAGTCGCCGTGCC |
| 18S-R | GCCTGCTGCCTTCCTTGG |
| TIFIA-F | ATTTTGTGCCTCCCCGAGTG |
| TIFIA-R | AGAAACCACGGTGTCGATGG |
| siRNAs | |
| siRNA name | Sequence |
| siRNA-circERBB2-1 | CCUGGAUAUCAGGGACAGGUU |
| siRNA-circERBB2-2 | GGAUAUCAGGGACAGGCAGUU |
| siRNA-PA2G4-1 | GGAUUAUAUUCUCAAGGAAGG |
| siRNA-PA2G4-2 | CCACGGAAGACUACUUUAAAU |
| siRNA-TIFIA-1 | GCUGUUAGAUCCAGACAUAAA |
| siRNA-TIFIA-2 | GGAAGGCGAUGUAGAUGUUUC |
| CRISPR-cas9 gRNAs | |
| gRNA-3’ALU-5’ | AAAGCTCTTAGAACGGTGCCTGG |
| gRNA-3’ALU-3’ | TCTTGAGTAGGTGGAACTACAGG |
| Desthiobiotin-labeled circERBB2 antisense DNA probe (RNA pulldown): | |
| GAGCACGTAGCCCTGCACCTCCTGGATATCAGGGACAGGCAGTCACACAGCTGGCGCCGA | |
| biotin-labeled circERBB2 antisense RNA probe (FISH): | |
| GGGACCUGCCUCACUUGGUUGUGAGCGAUGAGCACGUAGCCCUGCACCUCCUGGAUAUCA  GGGACAGGCAGUCACACAGCUGGCGCCGAAUGUAUACCGGCCCUCGGGA | |

**Table S2: Critical reagent**

| Reagent name | Resource | Cat No. |
| --- | --- | --- |
| Antibody | | |
| Ki67 | Abcam | ab15580 |
| NCL | Abcam | ab129200 |
| PA2G4 | Abcam | ab180602 |
| β-actin | Sigma | A1978 |
| TIFIA | Santa | sc-390464 |
| RPA194 | Santa | sc-48385 |
| Goat anti rabbit secondary antibody, Alexa Fluor 594 conjugate | Abcam | ab150080 |
| Goat anti mouse secondary antibody, Alexa Fluor 488 conjugate | Abcam | ab150113 |
| Goat anti rabbit secondary antibody, HRP conjugate | Sigma | SAB3700885 |
| Goat anti mouse secondary antibody, HRP conjugate | Sigma | SAB3701084 |
| Chemicals and kits | | |
| RNase R | Geneseed | R0301 |
| Sybr Green | Yeasen | 11202ES03 |
| RevertAid First Strand cDNA Synthesis Kit | Thermo | K1622 |
| Cell Count Kit-8 | Yeasen | 40203ES60 |
| Dual Luciferase Reporter Assay Kit | Yeasen | 11402ES60 |
| Magnetic RNA-protein Pull-down Kit | Thermo | 20164 |
| Protease inhibitor | Yeasen | 20113ES08 |
| Protease K | Yeasen | 10401ES60 |
| RNase inhibitor | Beyotime | R0102 |
| Flag Beads | Sigma | F2220 |
| Seamless Clone Kit | Beyotime | D7010S |
| Lipofectamine 2000 | Invitrogen | A7592 |
| Silver stain kit | Beyotime | P0017S |
| Fetal Bovine Serum | Sigma | F9423 |
