# Supplementary figures and images for: Circular RNA circERBB2 promotes gallbladder cancer progression by regulating PA2G4-dependent rDNA transcription
Source: Mol Cancer. 2019 Nov 21;18:166. doi: 10.1186/s12943-019-1098-8 (PMC6868820; doi:10.1186/s12943-019-1098-8)

a

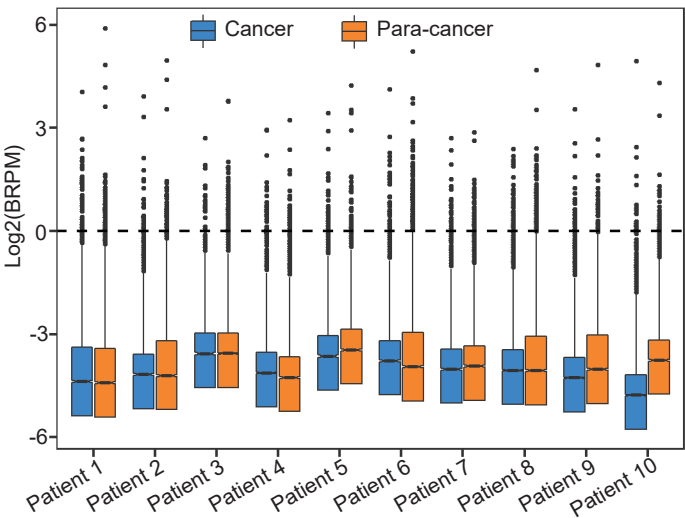

b

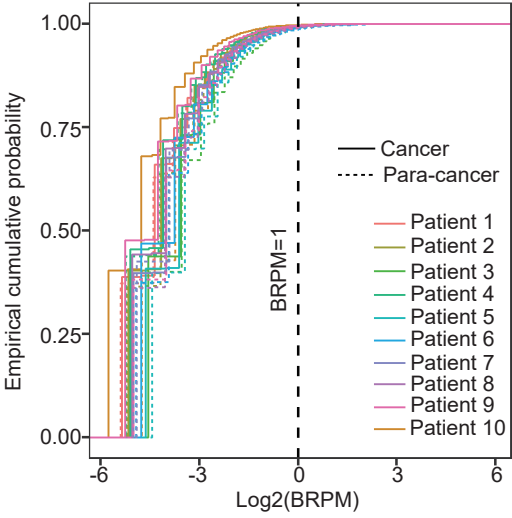

c

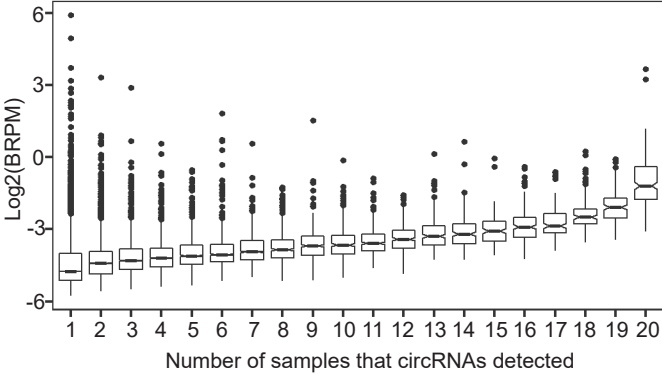

d

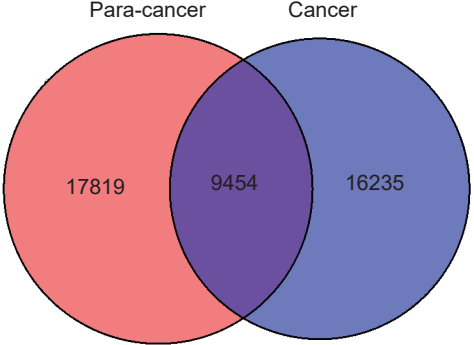

e

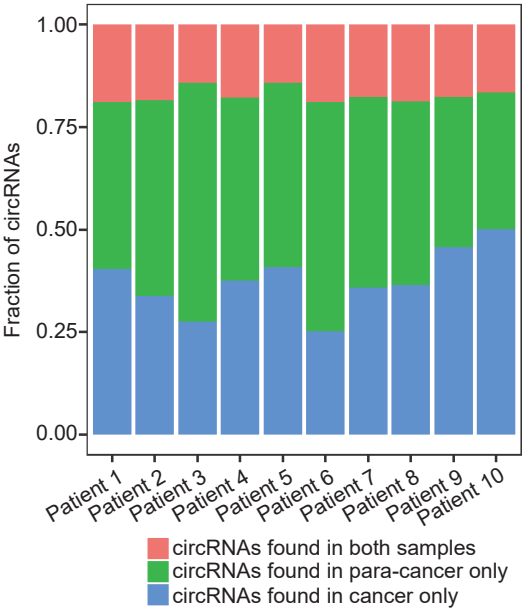

f

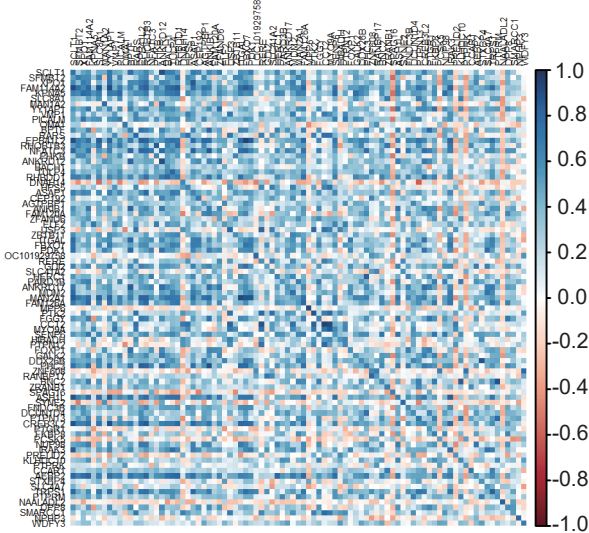

Supplement: Supplementary file 2 — Additional file 2: Figure S1. Different circRNA expression patterns between GBC and para-cancer tissues. a Expression levels of different circRNAs varied tremendously, BRPM values of different circRNAs ranged from less than 0.1 to more than 100. b Most circRNAs expressed at very low level, with more than 95% circRNAs had BRPM< 1. c circRNAs that detected in more samples tended to have higher expression level. d Only about 20% of circRNAs were detected in both cancer and para-cancer tissues. e For each patient, only 14 to 20% circRNAs were found in both cancer and para-cancer tissues. f circRNAs that generated from same gene locus tended to had positive correlation with each other. The color scale indicates the value of Pearson correlation. [file 12943_2019_1098_MOESM2_ESM.pdf]

a

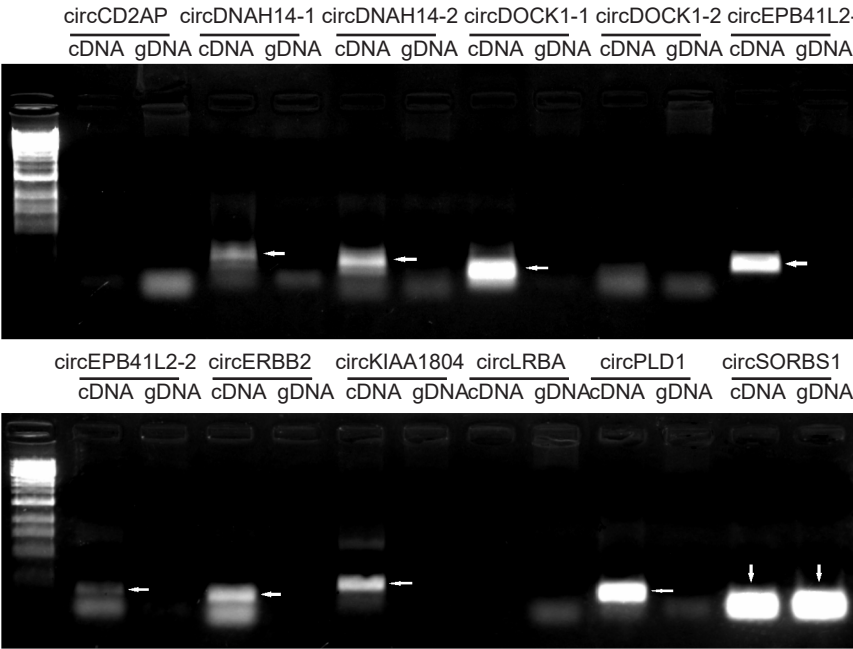

b

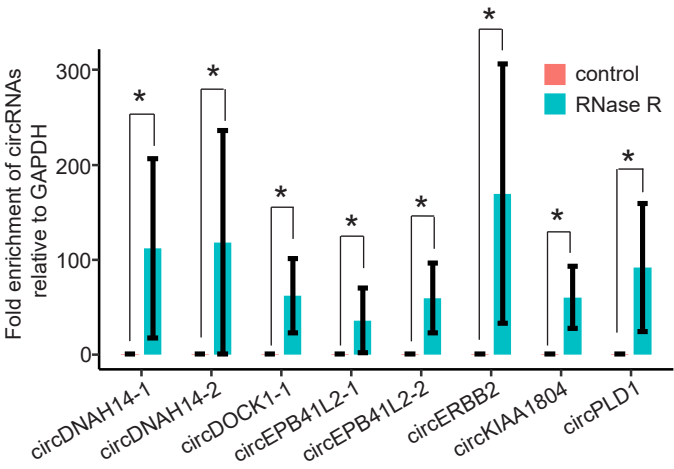

Supplement: Supplementary file 3 — Additional file 3: Figure S2. Screen for functional circRNAs in GBC tissues. a PCR validation of 12 circRNAs with divergent primers. cDNA or gDNA from SGC-996 cells were used as template. Arrows showed bands detected with back-splicing sequence by DNA sequencing. b Relative abundance of the 8 circRNAs increased significantly after treatment of RNase R (n = 3). Quantitative data from three independent experiments was presented as mean ± SD (error bars). P-values were determined by paired, two-tailed two sample t-test. *: p < 0.05. [file 12943_2019_1098_MOESM3_ESM.pdf]

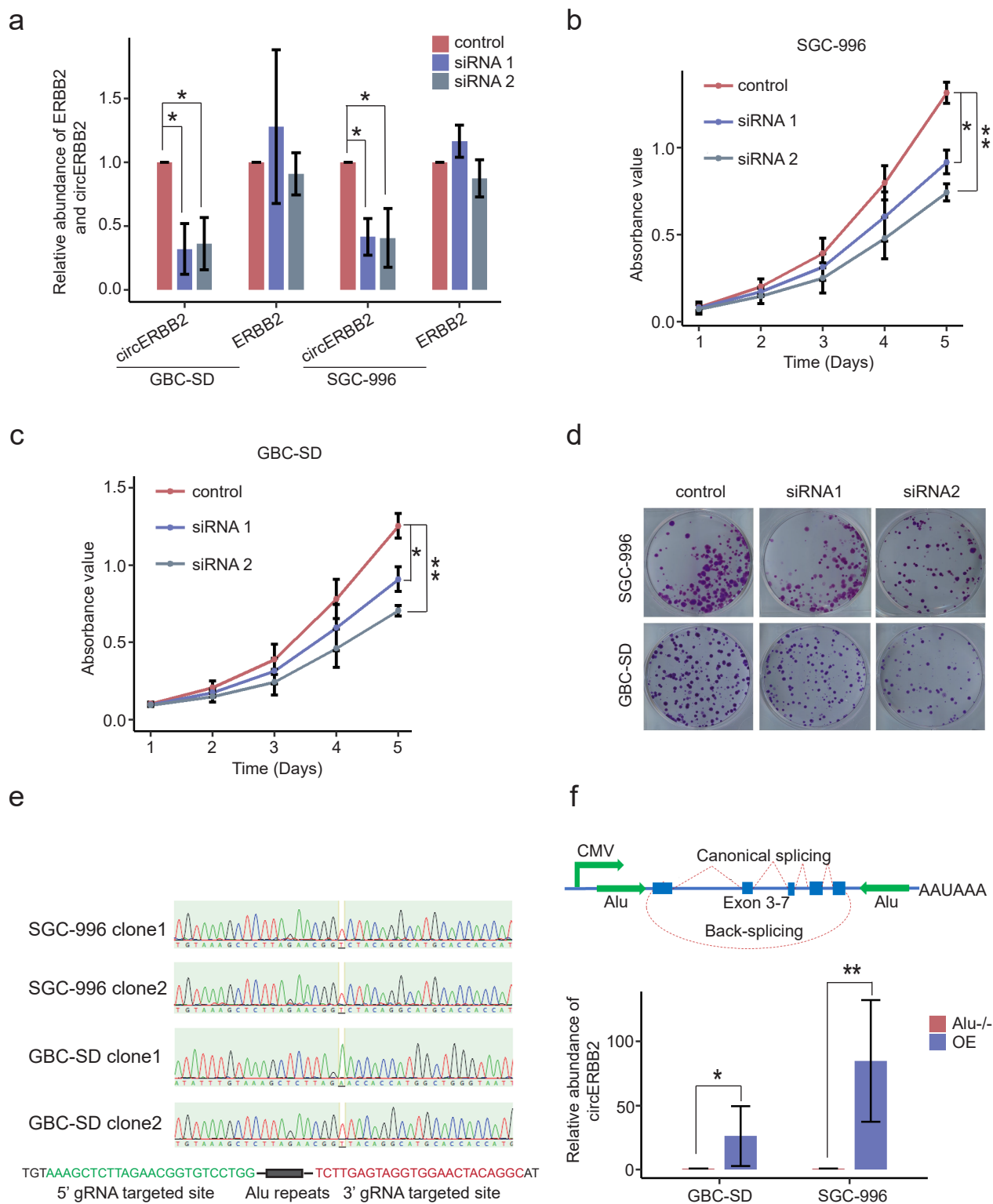

Supplement: Supplementary file 4 — Additional file 4: Figure S3. circERBB2 promotes growth of GBC cells in vitro. a qPCR showed that circERBB2, but not ERBB2 mRNA, was effectively silenced by two siRNAs targeting back-splicing sequence of circERBB2 (n = 3). b CCK8 assay showed that silencing of circERBB2 by siRNAs impaired proliferation of SGC-996 cells (n = 3). c CCK8 assay showed that silencing of circERBB2 by siRNAs impaired proliferation of GBC-SD cells (n = 3). d Silencing of circERBB2 by siRNAs impaired clone formation ability of GBC cells. e gDNA sequence of sgRNA-targeted region of SGC-996Alu−/− and GBC-SDAlu−/− cells. f Structure of pLenti-CMV-circERBB2 vector and relative abundance of circERBB2 in GBC cells with or without OE of circERBB2 (n = 3). Quantitative data from three independent experiments was presented as mean ± SD (error bars). P-values were determined by paired, two-tailed two sample t-test. *: p < 0.05; **:p < 0.01. [file 12943_2019_1098_MOESM4_ESM.pdf]

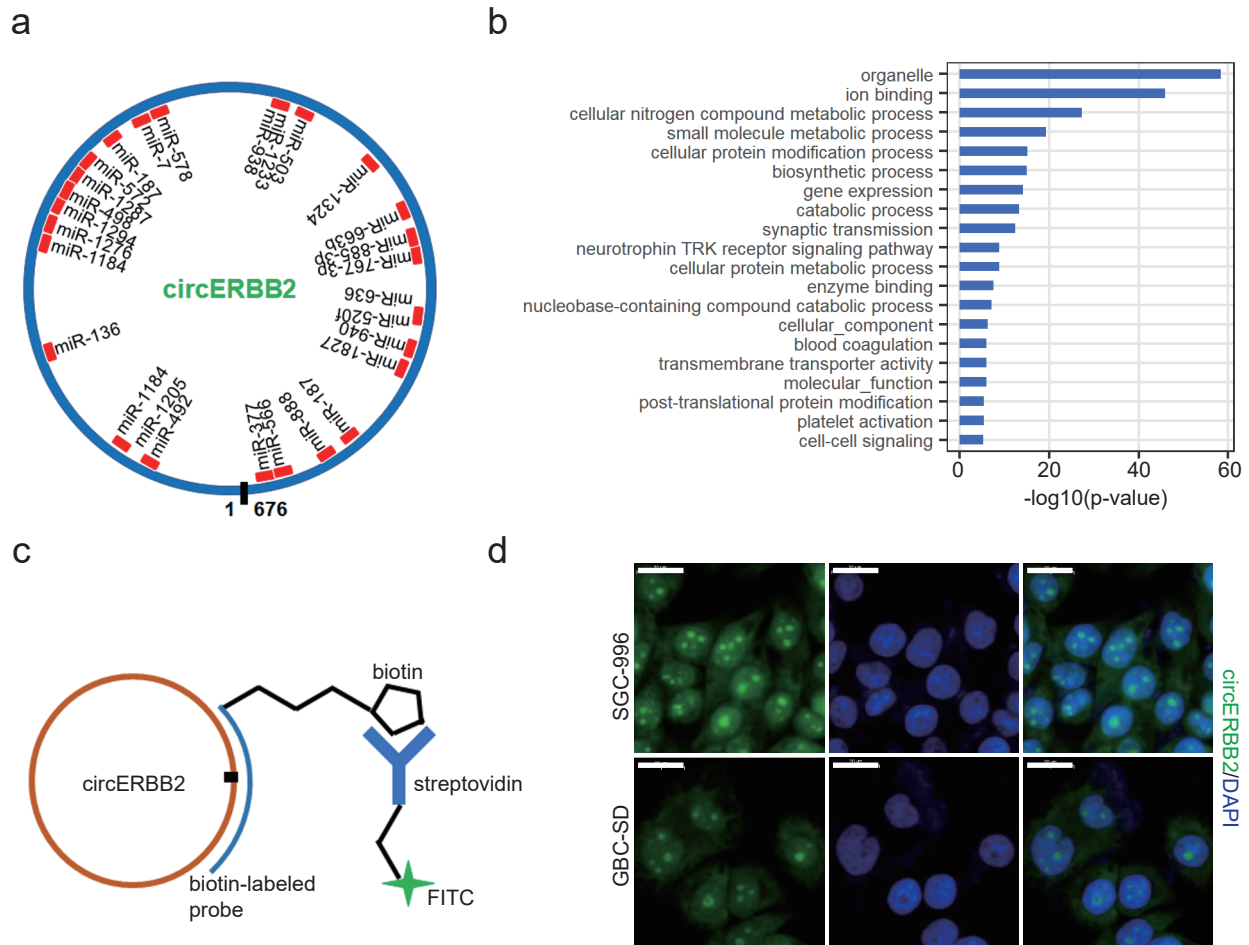

Supplement: Supplementary file 5 — Additional file 5: Figure S4. Nucleolar localization of circERBB2. a Schematic plot showed miRNAs that predicted as targets of circERBB2 by using circular RNA Interactome. b GO analysis of genes targeted by those miRNAs, and the results were unrelated with cellular proliferation. c Schematic plot of FISH assay with biotin-label RNA probe targeting back-splicing sequence of circERBB2. d FISH assay revealed sub-cellular localization of circERBB2. Scale bar: 20 μm. [file 12943_2019_1098_MOESM5_ESM.pdf]

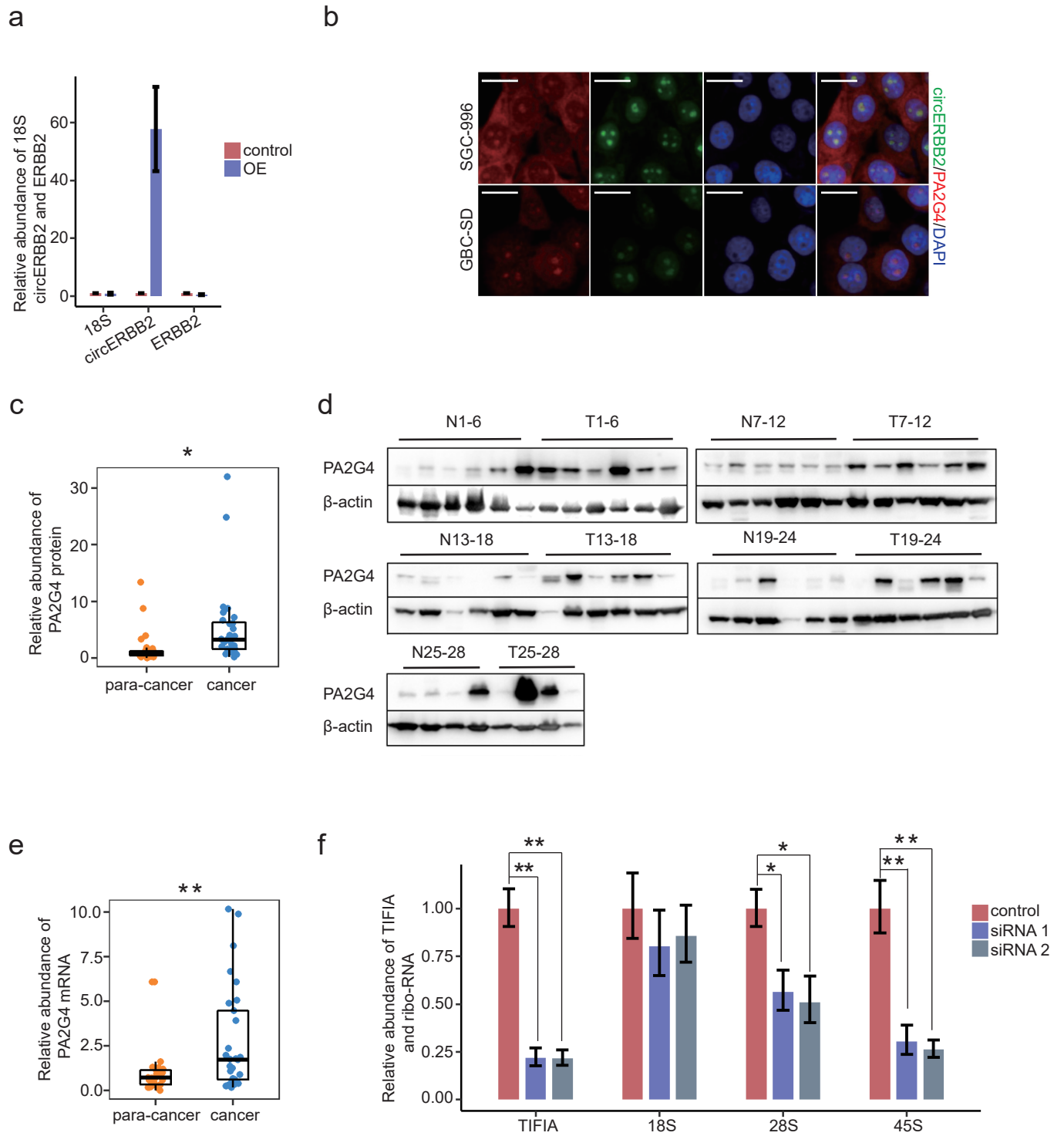

Supplement: Supplementary file 6 — Additional file 6: Figure S5. circERBB2 interacts with PA2G4. a qPCR showed that desthiobiotin-labeled DNA probe effectively captured circERBB2 (n = 3). b FISH+IF double staining showed that both circERBB2 and PA2G4 was accumulated in the nucleolus. Scale bar, 5 mm. c, d Western blot showed that PA2G4 protein increased significantly in GBC tissue, compared with para-cancer tissues (n = 28). e PA2G4 mRNA increased significantly in GBC tissues, compared with para-cancer tissues (n = 29). f qPCR showed silencing of TIFIA with two siRNAs severely impaired rDNA transcription and rRNA genesis in GBC cells (n = 3). Quantitative data from three independent experiments was presented as mean ± SD (error bars). P-values were determined by paired, two-tailed two sample t-test. *:p < 0.05; **:p < 0.01. [file 12943_2019_1098_MOESM6_ESM.pdf]

a

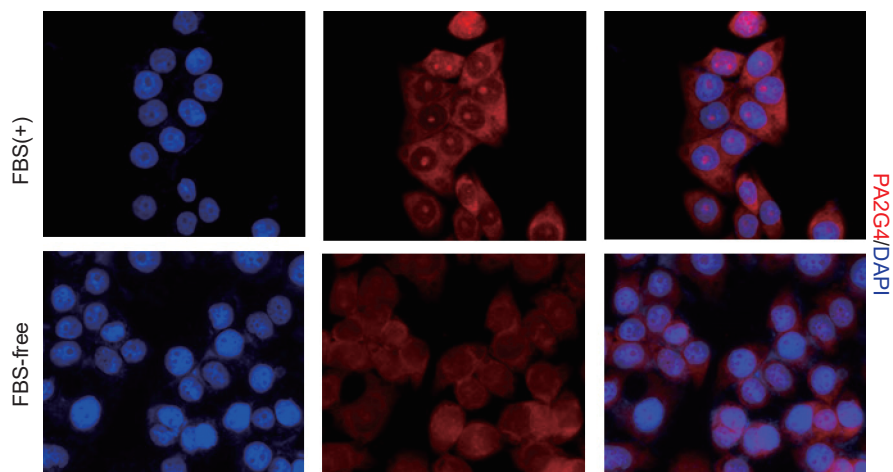

b

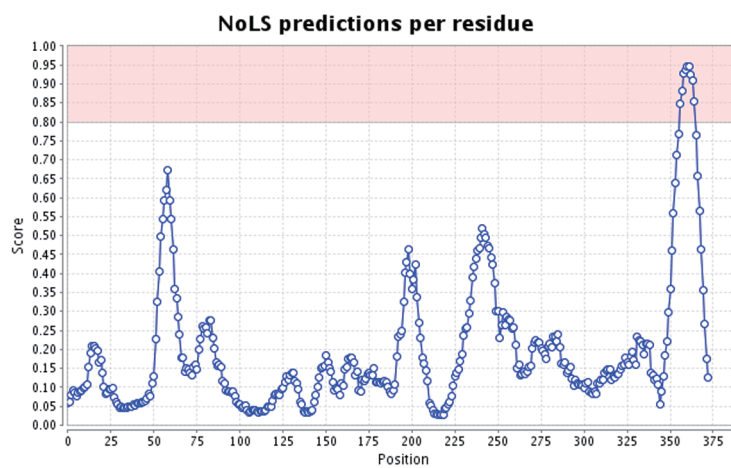

Supplement: Supplementary file 7 — Additional file 7: Figure S6. circERBB2 regulates nucleolar-localization of PA2G4. a IF showed nucleolar localization of PA2G4 was decreased when SGC-996 cells were cultured in FBS-free medium. b Screen for nucleolar localization sequence of PA2G4 with NoD. [file 12943_2019_1098_MOESM7_ESM.pdf]
